# Corrosion‐Resistant Ultrathin Cu Film Deposited on N‐Doped Amorphous Carbon Film Substrate and Its Use for Crumpleable Circuit Board

**DOI:** 10.1002/advs.202403587

**Published:** 2024-08-29

**Authors:** Chae‐Eun Shim, Sangseob Lee, Minsik Kong, Ik‐Soo Kim, Jaeik Kwak, Woosun Jang, Se‐Young Jeong, Dong Wook Kim, Aloysius Soon, Unyong Jeong

**Affiliations:** ^1^ Department of Materials Science and Engineering Pohang University of Science and Technology (POSTECH) Pohang 37673 Republic of Korea; ^2^ Department of Materials Science and Engineering and Center for Artificial Synesthesia Materials Yonsei University Seoul 03722 Republic of Korea; ^3^ Department of Integrated Science and Engineering Division Underwood International College Yonsei University Incheon 21983 Republic of Korea; ^4^ Gordon Center for Medical Imaging Department of Radiology Massachusetts General Hospital and Harvard Medical School Boston MA 02114 USA; ^5^ Physical Intelligence Department Max Planck Institute for Intelligent Systems 70569 Stuttgart Germany

**Keywords:** amorphous carbon, anti‐corrosion, copper electrode, flexible circuit board, nitrogen‐doping

## Abstract

Copper (Cu) is widely used as an industrial electrode due to its high electrical conductivity, mechanical properties, and cost‐effectiveness. However, Cu is susceptible to corrosion, which degrades device performance over time. Although various methods (alloying, physical passivation, surface treatment, etc.) are introduced to address the corrosion issue, they can cause decreased conductivity or vertical insulation. Here, using the nitrogen‐doped amorphous carbon (*a‐C*:N) thin film is proposed as a substrate on which Cu is directly deposited. This simple method significantly inhibits corrosion of ultrathin Cu (<20 nm) films in humid conditions, enabling the fabrication of ultrathin electronic circuit boards without corrosion under ambient conditions. This study investigates the origin of corrosion resistance through comprehensive microscopic/spectroscopic characterizations and density‐functional theory (DFT) calculations: i) diffusion of Cu atoms into the *a‐C*:N driven by stable C‐Cu‐N bond formation, ii) diffusion of N atoms from the *a*‐C:N to the Cu layer heading the top surface, which is the thermodynamically preferred location for N, and iii) the doped N atoms in Cu layer suppress the inclusion of O into the Cu lattice. By leveraging the ultrathinness and deformability of the circuit board, a transparent electrode and a crumpleable LED lighting device are demonstrated.

## Graphical abstract

Through the synergistic effect of the strong C‐Cu‐N bond formation facilitated by the diffused Cu in the N‐doped amorphous carbon (*a‐C*:N) and the inhibition of Cu vacancy formation by the diffused N in the Cu layer, ultrathin Cu films deposited on the *a‐C*:N film substrate are corrosion‐resistant. The Cu/*a‐C*:N thin film is used as an electronic circuit board for crumpled devices.

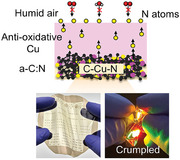

## 1 Introduction

Cu has been a widely preferred option for electrodes in the industry due to its excellent electrical and thermal conductivities, relatively soft mechanical properties, and lower cost compared to noble metals. Unfortunately, Cu is susceptible to corrosion when exposed to moisture in the air, which causes degradation of device performance over time.^[^ ^1^, ^2^, ^3^, ^4^ ^]^ Very recently, it has been revealed that a single crystal Cu film with atomic‐level surface roughness can have a positive energy barrier against oxidation, ensuring the stability of the Cu thin film in atmospheric conditions.^[^ ^5^, ^6^ ^]^ However, the limited feasibility of producing single‐crystal films restricts its widespread application in electronic device fabrication.

So far, various approaches have been explored to prevent or minimize the corrosion of Cu,^[^ ^1^ ^]^ including metal alloy formation,^[^ ^7^, ^8^, ^9^ ^]^ physical passivation,^[^ ^10^, ^11^, ^12^, ^13^, ^14^, ^15^, ^16^ ^]^ and surface treatment.^[^ ^1^, ^2^, ^17^, ^18^, ^19^ ^]^ Metal alloying or metal doping involves adding specific metals (Zn, Sn, Si, Ni, Al, etc.) to the Cu lattice to reduce the Cu ion mobility, thereby blocking oxygen reduction on Cu. Some alloying elements in Cu alloys promote the formation of a protective oxide layer on the surface, acting as a barrier against further corrosion.^[^ ^8^, ^20^ ^]^ Alloy formation typically decreases the electrical conductivity of Cu and increases its hardness,^[^ ^7^, ^8^, ^9^ ^]^ which is not preferred in an electrode, especially for futuristic flexible electronics. Physical passivation has been extensively attempted by covering the Cu surface with a 2D conductive material (such as graphene and graphene oxide)^[^ ^16^ ^]^ or a 2D non‐conductive material (such as h‐BN and Si_3_N_4_).^[^ ^14^, ^15^ ^]^ The conductive 2D layer, especially single‐crystal graphene, acts as an effective barrier against oxygen penetration.^[^ ^16^ ^]^ However, the presence of inevitable defects (holes, cracks, and grain boundaries) is known to accelerate corrosion because the formation of a galvanic cell through the conductive barrier leads to electrochemical reactions.^[^ ^11^, ^21^, ^22^ ^]^ Although covering with non‐conductive 2D materials can prevent galvanic corrosion,^[^ ^14^, ^15^ ^]^ electrical interconnection through the passivation layer remains a major drawback. In addition, transferring the 2D passivation layer suffers from incomplete transfer, a complicated procedure, and weak adhesion to the Cu layer.^[^ ^23^, ^24^, ^25^, ^26^, ^27^, ^28^, ^29^ ^]^ Superhydrophobic surface treatments on the Cu film can reduce the diffusion of water molecules,^[^ ^2^, ^17^ ^]^ and a coating layer containing corrosion inhibitors (such as azoles, amines, thiols, amino acids, etc.) impede corrosion by intercepting oxygen or reacting with oxygen.^[^ ^1^, ^2^, ^18^, ^19^ ^]^ Post‐treatment for incorporating N atoms on the Cu surface has also been applied to address the corrosion issue, however, the post‐treatment led to surface etching and degraded the quality of ultrathin Cu films.^[^ ^30^, ^31^ ^]^ Due to these technological challenges, it is crucial to preserve the inherent electrical properties of a Cu thin film for use in ultrathin devices. Considering the pros and cons of the previous approaches, a desired method to prevent or minimize Cu corrosion is the direct deposition of Cu on a nanoporous substrate containing corrosion inhibitors. The nanopores are expected to allow diffusion of Cu through the substrate, which stabilizes the substrate‐Cu interfaces and maximizes the contact between corrosion inhibitors with the deposited Cu. Since the Cu layer fabricated by this approach does not require post‐treatment and can be used for electric circuit lines through the conventional deposition process.

This study aims to investigate a practical method for stabilizing an ultrathin Cu film (<20 nm) deposited on an ultrathin N‐doped amorphous carbon (*a*‐C:N) substrate. We chose amorphous carbon owing to its nanopores and easy incorporation of N atoms in the entire body of the film. The method is based on the migration of Cu into the *a*‐C:N film substrate, the formation of stable bonds, and the potential variation of Cu surface energy in the presence of nitrogen in the substrate. Through detailed experimental analysis and density functional theory (DFT) calculations, we have discovered that the stable C‐Cu‐N bond facilitates the diffusion of Cu into the *a‐C*:N film. This process enhances adhesion to the Cu layer and allows for the creation of a continuous ultrathin Cu film (4 nm in thickness). We also reveal that the N atoms diffuse onto the Cu top surface and play a crucial role in retarding corrosion. We demonstrate a crumpleable circuit board by using the Cu/*a‐C*:N as an ultrathin flexible electrode without corrosion.

## 2 Results and Discussion

### 2.1 Characterization of the Cu Film Deposited on the *a‐C*:N Substrate

The *a‐C*:N films were produced on Si wafers by modifying our reported procedure, which is based on the one‐step, microwave‐assisted thermal decomposition of a spin‐coated branched polyethyleneimine (b‐PEI) thin film.^[^ ^32^ ^]^ D‐(+) glucose was added to the PEI solution to improve the uniformity of the spin‐coated polymer film and facilitate caramelization during the subsequent thermal baking process. In this study, we fixed the weight ratio of PEI to glucose at 2:1 because this mixture ensured the uniformity of the polymer film, resulting in a homogeneous carbon film with a root mean square roughness (R_q_) of 0.19 nm (Figure S1, Supporting Information). From X‐ray photoelectron spectroscopy (XPS), the atomic percentage of N (at%) in the *a‐C*:N film was ≈23 at%, slightly lower than the at% of N in the polymer film (Figure S2, Supporting Information). The thickness of the *a‐C*:N film could be controlled from 2 to 30 nm,^[^ ^33^ ^]^ depending on the polymer film thickness. We fixed the *a‐C*:N film thickness at 10 nm in this study to ensure high transparency of the *a‐C*:N film substrate (Figure S3, Supporting Information). From spectroscopic analysis (XPS, elemental mapping, Raman spectroscopy) and high‐resolution transmission electron microscopy (HRTEM) of the *a‐C*:N film (Figure S4, Supporting Information), it was observed that the distribution of N in the *a‐C*:N film was homogeneous. The N atoms were found to exist in the forms of pyridinic and pyrrolic bond structures rather than the graphitic structure. Cu has been known to form strong coordination with N atoms in carbonaceous materials, especially with the pyridinic‐N and pyrrolic‐N groups, which are known as Cu‐philic.^[^ ^34^, ^35^, ^36^, ^37^ ^]^

**Figure 1:**
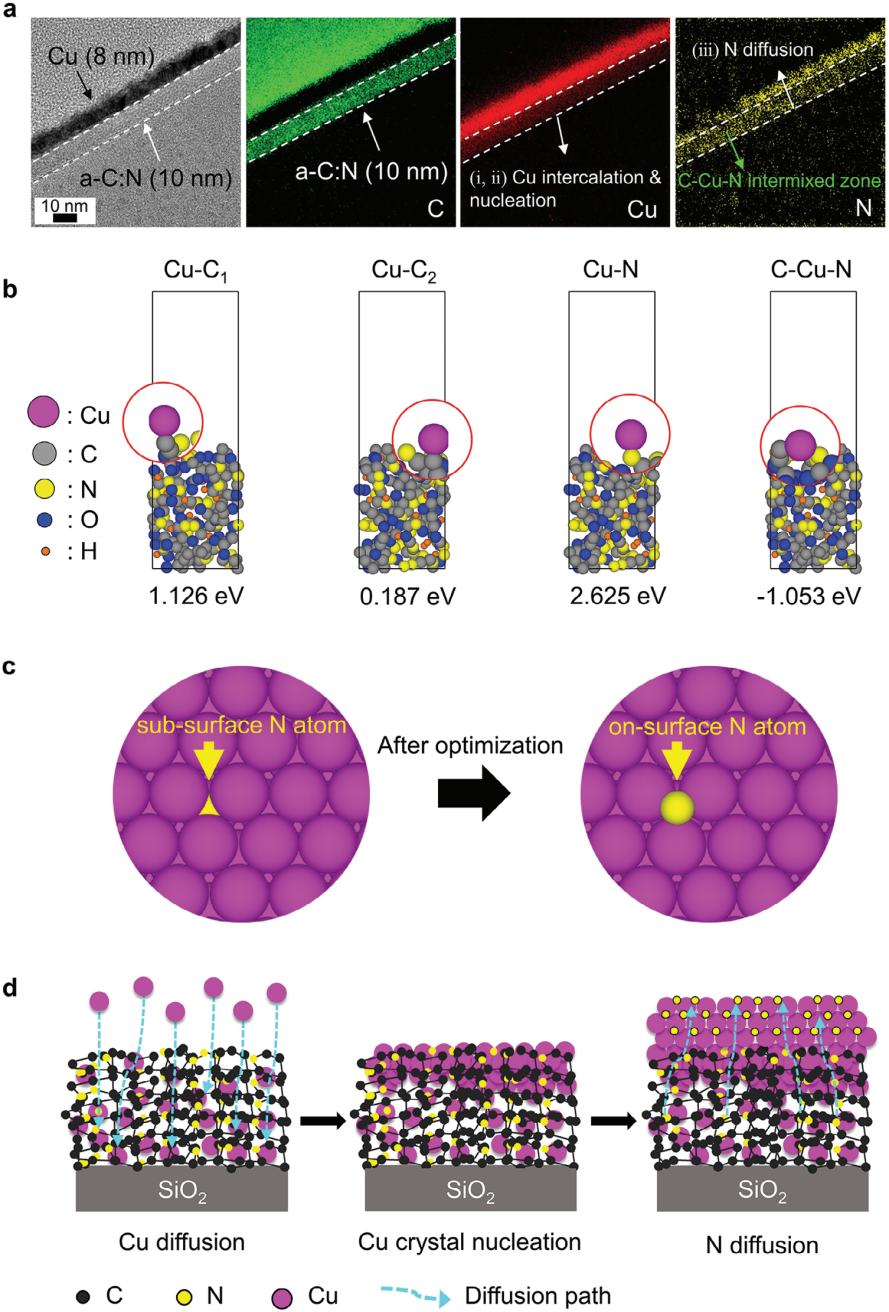
Diffusion of Cu into the *a‐C*:N film and diffusion of N into the Cu deposition layer. a) Cross‐sectional transmission electron microscope (TEM) image and its electron energy loss spectroscopy (EELS) mapping of the Cu (8 nm)/*a‐C*:N film, showing elemental mapping for C, Cu, and N. b) Side view of the atomic structures and energy for Cu adsorption on the *a‐C*:N slab model for Cu‐C_1_, Cu‐C_2_, Cu‐N, and C‐Cu‐N bonds. c) Top view of the atomic structure for the position of an N atom in a Cu(111) slab. Cu and N atoms are depicted in magenta and yellow, respectively. d) Scheme of the diffusion process, depicting i) Cu diffusion and C‐Cu‐N bond formation in the *a‐C*:N film, ii) nucleation and growth of continuous Cu surface layer due to the good wettability, iii) diffusion of N from the *a‐C*:N film toward the surface of Cu film during the Cu layer deposition.

Using thermal evaporation, we deposited Cu thin films on the *a‐C*:N film. **Figure** **1**a shows an electron energy loss spectroscopy (EELS) mapping for the Cu (8 nm)/*a*‐C:N bilayer cross‐section. A protective carbon layer was deposited on the bilayer film, and its cross‐section specimens were prepared using focused ion beam (FIB) milling. The EELS mapping revealed that Cu was uniformly distributed throughout the entire carbon layer. N was detected in both the Cu layer and the *a‐C*:N layer, while C was not found in the Cu layer. This result indicates that mutual diffusion of Cu and N occurred during the Cu deposition process. We obtained the same mutual distribution of Cu and N in the Cu (20 nm)/*a‐C*:N bilayer film (Figure S5, Supporting Information).

In order to uncover the origin of the Cu diffusion into the *a‐C*:N film, we compared the thermodynamic preferences of Cu for various atomic bonds. Classical molecular dynamics simulations (CMD) were performed to model the bulk *a‐C*:N structure. The corresponding radial distribution function of the bulk *a‐C*:N structure is shown in Figure S6 (Supporting Information). With the bulk *a‐C*:N structure, we generated a representative surface slab model and optimized the surface geometry using the first‐principles density functional theory (DFT). The adsorption energies of Cu at different binding sites are indicated as Cu‐C_1_, Cu‐C_2_, Cu‐N, and C‐Cu‐N configurations (Figure 1b). When Cu forms a bond with either one C or N atom, the adsorption energy of Cu on the *a‐C*:N surface slab was 1.126 eV for Cu‐C_1_ (primary), 0.187 eV for Cu‐C_2_ (secondary), and 2.625 eV for Cu‐N. When Cu forms bonds with both C and N simultaneously (C‐Cu‐N), the adsorption energy was −1.053 eV. Therefore, the C‐Cu‐N bonds are thermodynamically favorable and promote the diffusion of Cu into the *a‐C*:N film. The pyridinic and pyrrolic configurations surrounding the vacancies are considered to be the main routes for Cu diffusion.

It has been reported that N atoms in *a‐C*:N with a high atomic % of N (20∼30 at%) can readily escape from the carbon film at atmospheric pressure to relax the structural stress in the amorphous carbon.^[^ ^38^, ^39^, ^40^ ^]^ This previous result supports the possible bond breaking of N and its diffusion into the Cu layer. We investigated the preferred location of N in a Cu (111) surface slab (Figure 1c). First, we considered two sub‐surface positions: the octahedral site and the tetrahedral site. In the case of the tetrahedral site, during the DFT optimization process, the sub‐surface N atom relaxed to the on‐surface *hcp* site, and this process was determined to be non‐activated. For this case, the on‐surface adsorption site for N was considered more stable than other sub‐surface sites. Consequently, the N atoms are thermodynamically driven to migrate to the Cu surface. This result is in line with previous reports.^[^ ^41^ ^]^

Based on the above experimental and theoretical calculations, we categorized the Cu film deposition process into three stages, as illustrated in Figure 1d. At the initial deposition, Cu atoms diffuse into the *a‐C*:N film to form the energetically stable C‐Cu‐N bonds. Once the C‐Cu‐N bonds are created at the most active sites, the Cu atoms near the surface of the *a‐C*:N layer film begin to nucleate crystals. The Cu‐philic N groups in the *a‐C*:N film act as a wetting site, effectively reducing the nucleation energy barrier and ensuring the uniform deposition of Cu.^[^ ^34^, ^42^, ^43^ ^]^ At the final stage, the N atoms in the *a‐C*:N film diffuse onto the Cu layer during film growth. The reduced surface roughness (R_q_) of the Cu layer on the *a‐C*:N layer can facilitate the formation of a continuous Cu layer even at a low average thickness (Figure S7, Supporting Information) and significantly decrease the sheet resistance (R_s_) of the Cu film (Figure S8, Supporting Information). When the thickness (t_Cu_) of the Cu layer was 4 nm, R_s_ of the Cu/SiO_2_ was 34.4 ± 5 Mohm sq^−1^ (R_q _= 0.58 nm), whereas R_s_ of the Cu/*a‐C*:N was 382 ± 12 ohm sq^−1^ (R_q _= 0.25 nm). R_s_ of the Cu/graphene was 5.1 ± 0.2 kohm sq^−1^ (R_q _= 0.6 nm), which was attributed to the conductivity of the graphene. R_s_ of the Cu layer on the *a‐C* without N (Cu/*a‐C*) was 94.6 ± 9 Mohm sq^−1^ (R_q _= 0.48 nm), indicating that wetting of Cu was not significantly improved due to the absence of the stable chemical interactions, even though the *a‐C* layer could also serve as a permeable membrane for Cu diffusion.^[^ ^44^, ^45^ ^]^ The C‐Cu‐N binding in the Cu/*a‐C*:N played a crucial role in enhancing the wettability of Cu.^[^ ^42^, ^43^, ^46^, ^47^, ^48^ ^]^ It is noteworthy that when t_Cu_ = 20 nm, Cu formed a continuous layer regardless of the substrate,^[^ ^49^, ^50^ ^]^ thus their R_s_ became similar.

### 2.2 Retarded Corrosion of the Cu Thin Film on the *a‐C*:N Substrate

**Figure 2:**
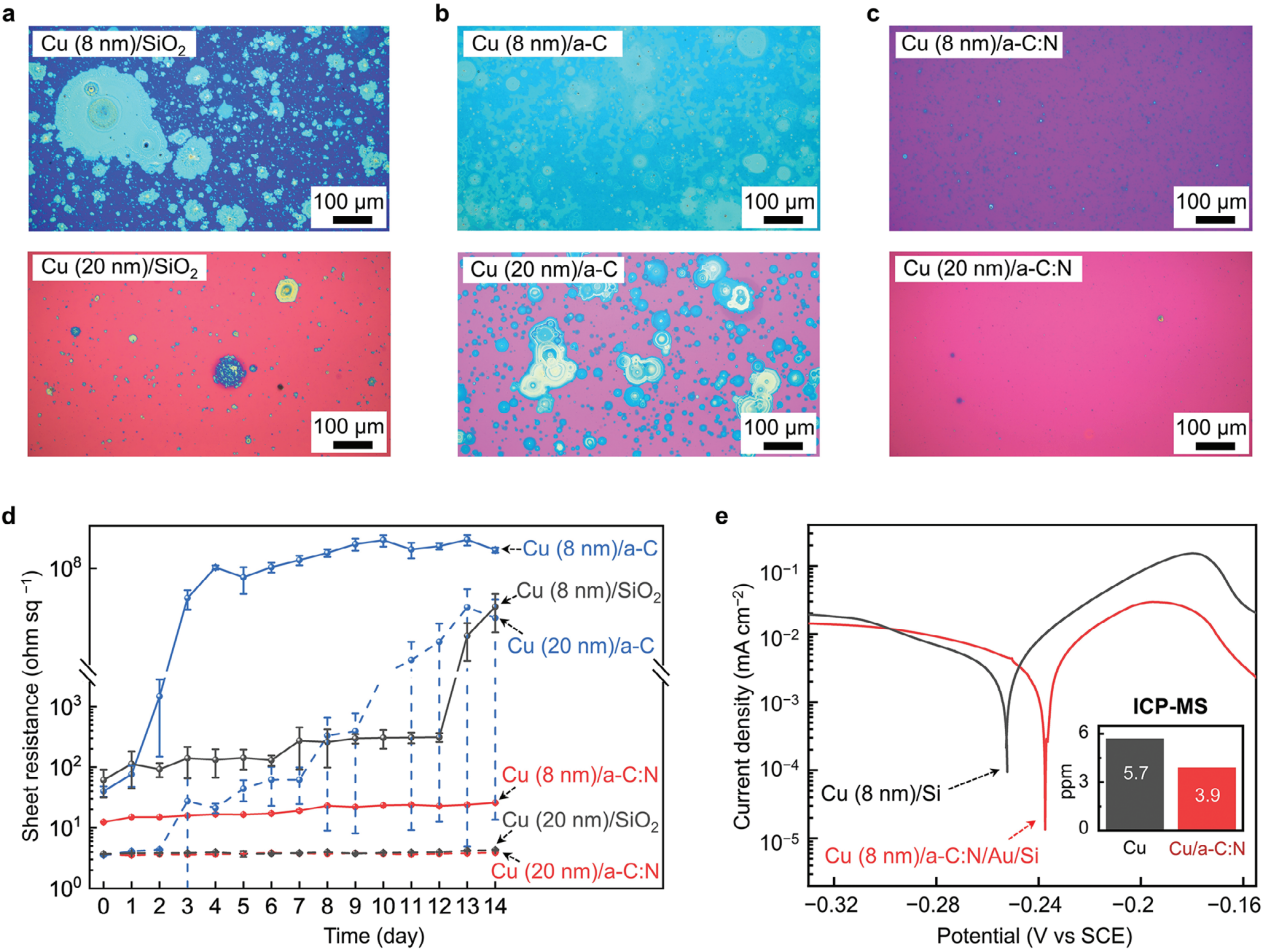
Corrosion of the Cu films on various substrates and the role of the *a*‐C:N film in corrosion resistance. a‐c) Optical microscope (OM) images of the corroded Cu films (8, 20 nm) on (a) SiO_2_, (b) *a‐C*, and (c) *a‐C*:N film after accelerated corrosion test. d) Changes in the sheet resistance (R_s_) during the accelerated corrosion test (14 days under humid conditions at 25 °C, 80% RH). e) Tafel polarization curves of the Cu (8 nm)/Si and the Cu (8 nm)/*a‐C*:N/Au/Si tested in a NaCl solution (3.5 wt.%). The inset bar graph shows the Cu ion concentration in the NaCl solution which was measured by inductively coupled plasma mass spectrometry (ICP‐MS).

Corrosion of the Cu film was examined using an optical microscope (OM) and by monitoring the changes in R_s_. The corrosion was accelerated under a humid environment (relative humidity (RH) = 80%) for 7 days at room temperature (25 °C). **Figure** **2**a–c compares the corrosion of the Cu/SiO_2_, Cu/*a‐C*, and Cu/*a‐C*:N films when the thickness (t_Cu_) of the Cu layer was 8 nm and 20 nm. Figure S9 (Supporting Information) shows the progression of corrosion after 1, 3, and 5 days of the corrosion test. On the Cu/SiO_2_ (Figure 2a) and the Cu/*a‐C* films (Figure 2b), pitting corrosion^[^ ^51^, ^52^ ^]^ was evident. This process led to the formation of localized cavities in the Cu layer due to anodic and cathodic reactions in the presence of water. It is noteworthy that the Cu/*a‐C* film showed accelerated corrosion compared with the Cu/SiO_2_ film. This was due to the porous *a‐C* substrate allowing water uptake, thereby facilitating corrosion from the Cu/*a‐C* interface in addition to the top Cu surface.^[^ ^43^, ^44^, ^45^ ^]^ On the contrary, the pitting corrosion in the Cu (8 nm)/*a‐C*:N film still remained localized, and the Cu (20 nm)/*a‐C*:N film exhibited only a limited number of localized pitting spots (Figure 2c).

Figure 2d and Table S1 (Supporting Information) compare R_s_ after the corrosion test for 14 days. Table S1 (Supporting Information) shows the daily numerical changes in R_s_. The R_s_ of the Cu (8 nm)/SiO_2_ film was large and unstable, while the R_s_ of the Cu (20 nm)/SiO_2_ film maintained its initial value due to the remaining electrical connection through the non‐oxidized Cu. After 13 days, the Cu (8 nm)/SiO_2_ was oxidized overall and exhibited insulating properties. The accelerated corrosion of the Cu/*a‐C* resulted in the Cu (8 nm) film, causing a significant increase in R_s_ of the Cu (20 nm) film after 3 days. After a week, the R_s_ of Cu (20 nm)/*a*‐C rapidly increased. The Cu/*a‐C*:N films almost maintained their R_s_ even in the Cu (8 nm) film after 2 weeks. When Cu was deposited on graphene, the oxidation of the Cu/graphene film was slower than that of the Cu/*a‐C* film but faster than the Cu/SiO_2_ film. As a result, the sheet resistance (R_s_) of the Cu (8 nm)/graphene film exceeded 2 kohm sq^−1^ after 2 days (Figure S10 and Table S2, Supporting Information), following the R_s_ of a graphene substrate due to the fully oxidized Cu layer. This suggests that carbon without strong binding with copper destabilized the copper film.

To confirm the corrosion stability of the Cu/*a‐C*:N film, we conducted an electrochemical test to obtain the polarization curves in a 3.5 wt% NaCl aqueous solution containing corrosive Cl^−^ ions. The polarization curves were obtained in independent anodic and cathodic scans starting around the open circuit potential (OCP). Three specimen sets (2 specimens in each set for anodic and cathodic scanning) were tested for this experiment, and we obtained reliable results. To remove the contact resistance issue between the Cu layer and the collector electrode, we used a heavily doped conductive Si wafer as a substrate after etching the native oxide layer of the wafer. For the Cu (8 nm)/Si, Cu was deposited on the conductive Si wafer and then placed on a current collector Cu plate for the electrochemical measurement. For the Cu (8 nm)/*a*‐C:N, we first deposited Au layer (50 nm) on the conductive Si wafer. Next, we generated the *a*‐C:N layer on the Au layer, covering the entire surface of Au/Si substrate. Subsequently, we deposited Cu on the *a*‐C:N, and finally placed the specimen on the current collector Cu plate. Au was deposited on the Si wafer to prevent the formation of the native oxide during the creation of the *a‐C*:N layer. Vertical electrical contact was assured with the measurement setup. It is notable that no electrochemical reaction took place between the NaCl electrolyte and the Si or Au substrate. Consequently, the polarization curves were generated from the reaction of the Cu layer during the anodic and cathodic scans. Figure 2e shows the Tafel polarization curves for the Cu (8 nm)/Si and the Cu (8 nm)/*a‐C*:N/Au/Si. The cathodic reaction involves the oxygen reduction reaction, while the anodic reaction involves the dissolution or oxidation of Cu. The decreasing current in the anodic reaction was due to the dissolution of Cu films (8 nm). The self‐corrosion potential (E*_corr_*) indicates the corrosion tendency.^[^ ^53^, ^54^, ^55^ ^]^ E*_corr_* values were obtained with logarithmic fitting by using NOVA software in the low current region. The averaged E*_corr_* for the Cu (8 nm)/*a‐C*:N/Au/Si was −0.238 ± 0.002 V (vs SCE) and −0.250 ± 0.006 V (vs SCE) for the Cu (8 nm)/Si. This difference in E*_corr_* was quantitatively compared by the Cu ion concentration in the NaCl solution measured by inductively coupled plasma mass spectrometry (ICP‐MS) (inset in Figure 2e). The Cu ion concentration from the Cu (8 nm)/Si and the Cu (8 nm)/*a‐C*:N/Au/Si was 5.7 ppm and 3.9 ppm, respectively, showing 30% less dissolution of Cu in the Cu (8 nm)/*a‐C*:N/Au/Si. From the results of E*_corr_* and ICP‐MS, the Cu (8 nm)/*a‐C*:N was less prone to electrochemical corrosion.

Figure S11 (Supporting Information) illustrates the Nyquist plots and Bode plots showing the impedance for each sample. The equivalent circuit consists of the solution resistance (R_sl_), the resistance of charge transfer for the reaction (R_1_), the resistance for ions to pass through the coated surface such as an oxide layer (R_2_), and their corresponding constant phase elements (CPEs). The CPEs indicate the imperfect capacitors, which are commonly used to analyze the corrosion of a metal thin film with a surface oxide layer. The Nyquist plot of the Cu (8 nm)/*a‐C*:N/Au/Si showed a larger radius than that of the Cu (8 nm)/Si. From Table S3 (Supporting Information), the solution resistance of the Cu (8 nm)/Si and the Cu (8 nm)/*a‐C*:N/Au/Si showed no electrical contact issue in this case. The sum of R_1_ and R_2_ corresponds to the resistance of the entire system where the corrosion reaction occurs, in which a higher value indicates higher corrosion resistance. Although R_1_ was similar for both cases, R_2_ of the Cu (8 nm)/*a‐C*:N/Au/Si (1120.2 ohm) was notably higher than R_2_ of the Cu (8 nm)/Si (99.4 ohm). On the basis of the electrochemical experiments, it is obvious that the N atoms on the surface of the Cu film impeded the access of corrosive anions such as O^2−^, OH^−^, and Cl^−^ in the corrosion medium.

### 2.3 Characterization of the Corroded Cu Films on the *a‐C*:N Substrate

**Figure 3:**
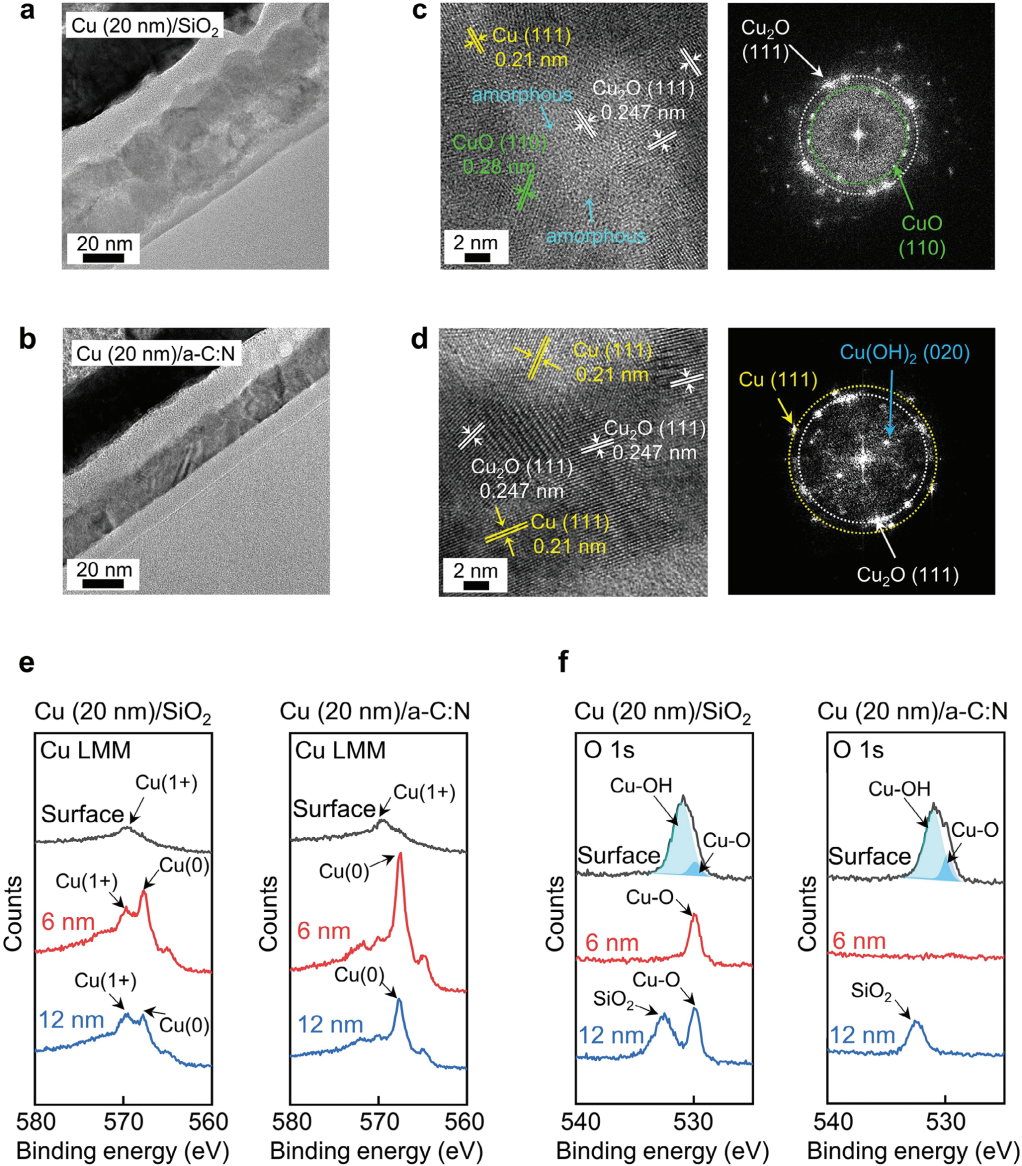
Characterization of the corroded Cu films on SiO_2_ and *a‐C*:N film. a‐d) Cross‐sectional TEM images and the corresponding HRTEM images with the fast Fourier transform (FFT) images; (a, c) low‐magnified TEM image and HRTEM image with FFT image for the corroded Cu (20 nm)/SiO_2_, and (b, d) for the oxidized Cu (20 nm)/*a‐C*:N (10 nm). e, f) X‐ray photoelectron spectroscopy (XPS) depth profile analysis in the Cu LMM (e) and the O 1s (f) regions collected from the corroded Cu (20 nm) on SiO_2_ and the *a‐C*:N substrate.

We examined the structural changes and chemical changes after the 7‐day corrosion test at RH = 80% (**Figure** **3**). The cross‐sectional TEM image of the Cu (20 nm)/SiO_2_ film (Figure 3a) revealed significant volume expansion (approximately 50 nm in thickness), whereas the Cu (20 nm)/*a‐C*:N film showed no noticeable volume increase (Figure 3b). The selected area electron diffraction (SAED) pattern of the oxidized Cu (20 nm)/SiO_2_ film revealed a significant amount of amorphous Cu oxides. In contrast, the SAED of the Cu (20 nm)/*a‐C*:N film indicated that the crystal structure of the metallic Cu was almost preserved (Figure S12, Supporting Information). The enlarged HRTEM images and their fast Fourier transform (FFT) patterns of the oxidized Cu layer on SiO_2_ are shown in Figure 3c and Figure S13a (Supporting Information). The apparent interplanar lattice spacing was 0.247 nm corresponding to Cu_2_O (111), and 0.28 nm corresponding to CuO (110), indicating the formation of Cu oxides.^[^ ^5^, ^56^ ^]^ Additionally, the halo ring in the FFT pattern suggests the amorphous nature of Cu oxides. The enlarged HRTEM image and the FFT pattern of the oxidized Cu/*a‐C*:N film (Figure 3d; Figure S13b, Supporting Information) revealed the preserved metallic Cu (111) and the addition of Cu_2_O (111) with the partial presence of Cu(OH)_2_ (020).

The changes in the crystal structure after the same corrosion test were also investigated using X‐ray diffraction (XRD) (Figure S14, Supporting Information). Initially, the Cu (20 nm)/SiO_2_ film exhibited a Cu_2_O (111) peak at 2*θ* = 37°, which was a result of the susceptibility to oxidation, even in the dry air environment of the X‐ray room. Following the 7‐day corrosion test at RH = 80%, the Cu (20 nm)/SiO_2_ film still showed the metallic Cu (111) peak, but it also exhibited a broad peak (2*θ* = 34−36°) corresponding to amorphous Cu oxides. This indicates that the initially formed Cu_2_O was further oxidized to Cu(OH)_2_ and CuO forms.^[^ ^57^ ^]^ This finding aligns with the amorphous nature of the oxide, as observed in the cross‐sectional TEM and SAED (Figure 3a,c). Comparatively, the Cu/*a‐C*:N film only exhibited the metallic Cu (111) peak initially, indicating that the film was stable under dry air conditions. After the oxidation process, a sharp Cu_2_O (111) peak (2*θ* = 37°) appeared, and the Cu (111) peak also became sharp. This suggests that grain growth of the metallic Cu grains occurred, and large Cu_2_O grains were formed during the oxidation process.

We investigated the depth profiles of Cu and O bonds using depth‐XPS analysis for the Cu (20 nm)/SiO_2_ and Cu (20 nm)/*a‐C*:N films in the oxidized specimen. From the Cu LMM binding energy spectra (Figure 3e), both films exhibited the oxidized Cu(1+) peak (569 eV) on the surface. At a depth of 6 nm from the surface, the Cu (20 nm)/SiO_2_ showed the coexistence of Cu(1+) and the metallic Cu(0) peak (568 eV), while the Cu/*a‐C*:N film predominantly displayed the Cu(0) peak (568 eV). At 12 nm depth, the Cu/SiO_2_ film still showed the coexistence of Cu(1+) and Cu(0), whereas the Cu/*a‐C*:N film only showed the Cu(0) peak. Regarding the O 1s binding (Figure 3f), both films showed the Cu‐OH peak (531 eV) and the Cu‐O (530 eV) peak on their surfaces. The atomic percentage of Cu‐OH was approximately 82% in the Cu/SiO_2_ film surface and 76% in the Cu/*a‐C*:N film surface, indicating that the humid environment caused the adsorption of hydroxides onto the surfaces. At 6 nm depth, the Cu/SiO_2_ film showed only Cu‐O bonds, while the Cu/*a‐C*:N film did not show any O binding, suggesting no oxidation within the Cu film. At 12 nm depth, the Cu/SiO_2_ film still exhibited the Cu‐O bond along with the Si‐O bond from the Si wafer, whereas the Cu/*a‐C*:N film only showed the Si‐O bond. It is noteworthy that the Cu/*a‐C* film showed the most prominent corrosion. The Cu(1+) bonding was evident on the surface, and only the Cu‐O bonding was observed at the depth of 12 nm owing to the volume expansion caused by severe corrosion (Figure S15, Supporting Information).

### 2.4 Corrosion Retardation Mechanism of the Cu/*a‐C*:N Film

**Figure 4:**
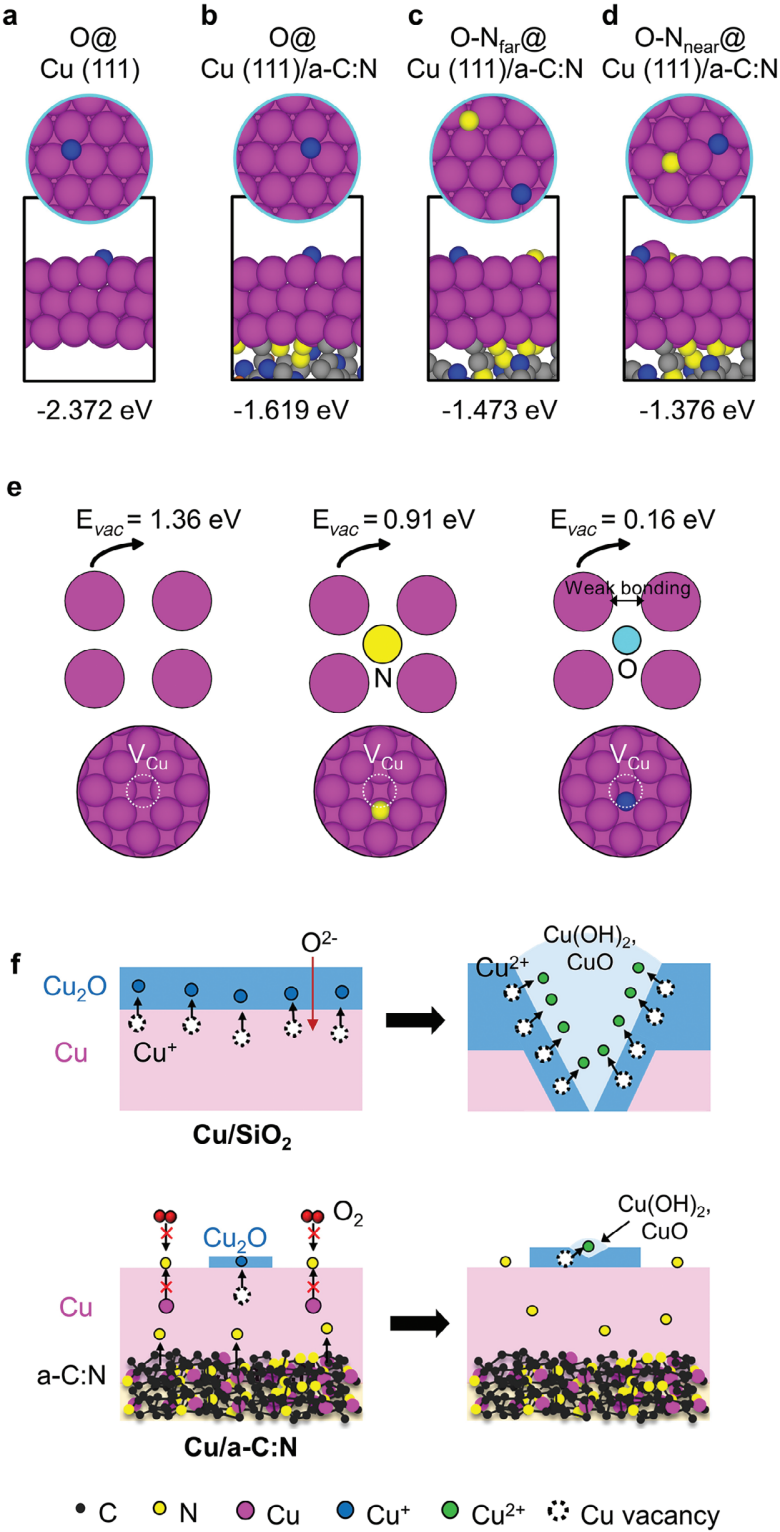
DFT simulations of O adsorption and Cu vacancy formation energy, along with a scheme of oxidation mechanism of Cu with/without the *a*‐C:N film. a‐d) Top and side views of the atomic structures with their energy for the O adsorption on the Cu(111) surface without and with the *a‐C*:N support; (a) O adsorption on Cu(111), (b) O adsorption on Cu(111)/*a‐C*:N, (c) O adsorption far away from N on the surface of Cu(111)/*a‐C*:N, and (d) O adsorption near to N on the surface of Cu (111)/*a‐C*:N models. e) Optimized atomic structures with their energy for the Cu vacancy formation energy without or with the N or O. f) Schemes for the Cu oxidation process without and with the *a*‐C:N.

The influence of the diffused N in the Cu layer on the oxidation process was investigated by comparing the adsorption energy of O atoms on Cu (111) without and with the *a‐C*:N bottom layer (**Figure** **4**a,b). In addition, with the *a‐C*:N bottom layer, we compared two adsorption surface models: one where both O and N are located away from the Cu (111) surface (Figure 4c) and another where the two atoms are nearby on the Cu surface (Figure 4d). The O adsorption energy on Cu (111) without the *a‐C*:N film was strongly exothermic (−2.372 eV), indicating that surface oxidation is spontaneous. The O binding energy was −1.619 eV with the *a‐C*:N bottom layer, thus the oxidation is less spontaneous than without the *a‐C*:N layer. The adsorption strength of the O atoms decreased further to −1.473 eV when the N atom was bound far away, and to −1.376 eV when the N atom was near the O adsorbate on the surface. The calculation implies a 40% reduction in oxygen affinity to Cu (111) when the *a‐C*:N film is used as a substrate. Therefore, our model suggests that the oxidation process can be effectively retarded.

It is known that the formation of Cu vacancies (V_Cu_) can increase the diffusive mobility of Cu atoms, accelerating the oxidation of Cu.^[^ ^57^, ^58^, ^59^, ^60^ ^]^ Hence, turning our attention to the bulk *fcc* Cu supercell model, we investigated the vacancy formation energy of Cu (E*_vac_*) in the bulk limit (Figure 4e). We positioned O and N atoms in the octahedral site because it is reported to be the most stable interstitial site for both O and N atoms.^[^ ^41^, ^61^ ^]^ E*_vac_* of bulk Cu was 1.36 eV. E*_vac_* was 0.91 eV when the N was near the vacancy site, whereas it was 0.16 eV when an O was near the vacancy site. This suggests that vacancy formation is facilitated when O is in proximity. The difference in the calculated E*_vac_* is due to the larger electronegativity of O (3.5) compared to N (3.0) because the O atom delocalizes and weakens the metallic Cu‐Cu bond. From the results of the wide N distribution in the Cu layer, it is reasonable to conclude that the inclusion of N decreases the rate of Cu vacancy formation and, consequently, the rate of oxidation.

Figure 4f schematically compares the oxidation process of a Cu film on a SiO_2_ and the *a‐C*:N substrate. According to the Cabrera‐Mott theory for metal oxidation in a humid air environment,^[^ ^59^, ^60^, ^62^, ^63^ ^]^ Cu_2_O is rapidly formed at the air/metal interface, which generates Cu ion vacancies, thus creating an electric field through the air/Cu_2_O interface. The electric field decreases the activation energy for further oxidation with O^2−^ and OH^−^ ions (Cu_2_O→Cu(OH)_2_ or CuO), which causes substantial Cu vacancies in the Cu_2_O layer. The aggressive migration of Cu ions causes a porous and uneven oxide layer, which is the reason for the observed amorphous oxide regions in this study. In the Cu/*a‐C*:N film, the N dopants on the Cu surface delay the adsorption of H_2_O and O_2_ due to the reduced O affinity to the Cu surface, thereby slowing down the transition from Cu to Cu_2_O. The strong C‐Cu‐N bond in the *a‐C*:N layer and the high E*_vac_* due to the N species in the Cu layer decrease the diffusivity of V_Cu_ or Cu ions. As a result, the further oxidation (Cu_2_O→Cu(OH)_2_ or CuO) can be effectively retarded.^[^ ^1^, ^2^, ^43^, ^47^ ^]^ This retardation mechanism leads to localized corrosion, while the rest of the film area remains resistant to oxidation.

### 2.5 Ultrathin Cu Circuit on the *a‐C*:N Substrate

The new generation of electrical devices requires electrodes and interconnections with high flexibility or transparency.^[^ ^64^, ^65^, ^66^ ^]^ Flexible transparent electrodes based on graphene,^[^ ^67^, ^68^ ^]^ carbon nanotubes (CNT),^[^ ^69^, ^70^ ^]^ conducting polymers,^[^ ^71^, ^72^, ^73^ ^]^ metal nanowires,^[^ ^74^, ^75^, ^76^ ^]^ and metal meshes^[^ ^77^, ^78^ ^]^ have been extensively investigated. Graphene, CNTs, and conducting polymer electrodes suffer from relatively low conductivity.^[^ ^67^, ^69^, ^79^ ^]^ Although large‐area metal electrodes made of metal nanowires and metal meshes have good conductivities, designing circuits with arbitrary shapes of electrodes and interconnections is not readily achieved. The coating process involved in the above‐mentioned electrodes does not allow for high‐resolution circuits and is not compatible with the contemporary industrial process of dry metal deposition. Therefore, the desirable approach to circuit design for flexible conductors is through vacuum deposition of an inexpensive thin metal circuit. The Cu deposition on the *a‐C*:N substrate meets the desirable approach.

**Figure 5:**
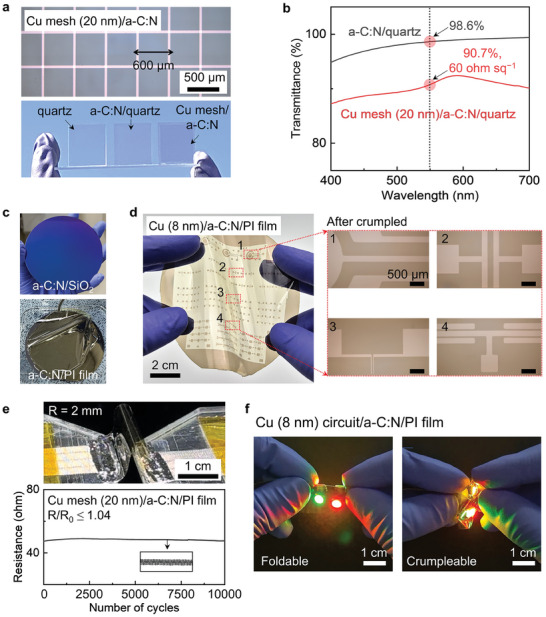
Ultrathin Cu/*a‐C*:N transparent electrode and crumpleable circuit board with mechanical and electrical stability. a) OM image of the Cu mesh (20 nm)/*a‐C*:N/quartz electrode (top) and photograph (bottom) of the bare quartz (left), *a‐C*:N/quartz (middle), and the Cu mesh (20 nm)/*a‐C*:N/quartz electrode (right). b) Transmittance spectra and sheet resistance of the *a‐C*:N/quartz and Cu mesh (20 nm)/*a‐C*:N/quartz electrode. c) Photographs of the *a*‐C:N prepared on the 4‐inch‐sized SiO_2_/Si wafer (top) and water‐assisted delamination onto the PI film (bottom). d) Photograph showing the fabrication of ultrathin Cu pattern on the flexible *a*‐C:N/PI substrate (left), and OM images of the Cu pattern (8 nm)/*a‐C*:N/PI after repeated crumpling. e) Photograph (top) of the transparent Cu mesh (20 nm)/*a‐C*:N/PI (15 µm) electrode during 10000 cyclic bending tests at R = 2 mm and its resistance change (bottom). f) Photographs of LED lighting at 2 V bias when the LEDs were attached to the ultrathin Cu (8 nm)/*a‐C*:N/PI film circuit board. Repeated folding (left) and crumpling (right) were exerted.

The *a‐C*:N film (10 nm in thickness) showed excellent optical transmittance (98.6% at 550 nm wavelength). We developed a transparent electrode featuring the thin Cu mesh on the *a‐C*:N film. This concept differs from the conventional Cu mesh transparent electrodes, which typically consist of narrow (a few micrometers) and thick (several hundred nanometers) metal lines with wide spacing (ranging from a few to thousands of micrometers).^[^ ^77^, ^78^, ^80^ ^]^ In our approach, we increased the metal line width (W_Cu_ = 50 µm, space = 500 µm) and reduced the thickness to 20 nm (**Figure** **5**a). The thin mesh electrode, produced by direct Cu deposition, showed high transmittance (90.7% at 550 nm) and low R_s_ (60 ± 8 ohm sq^−1^) (Figure 5b).

The *a‐C*:N film can be synthesized over a large area and easily transferred onto the polyimide (PI) film (Figure 5c). We fabricated the flexible ultrathin (8 nm) Cu pattern by depositing Cu on the *a‐C*:N/PI (15 µm) through a metal mask (Figure 5d). Even when crumpled, no cracks were visible on the patterns (inset of Figure 5d). To confirm the mechanical stability of the ultrathin Cu circuit on the flexible *a‐C*:N substrate, we conducted a cyclic bending test and a demonstration using light‐emitting diodes (LEDs). For the mechanical durability and oxidation stability of the transparent Cu mesh (20 nm)/*a‐C*:N/PI electrode, a bending test of 10000 cycles was performed at a 2 mm bending radius. The initial resistance (R_0_) of the mesh electrode was maintained during the bending cycles (Figure 5e), without showing any corrosion phenomenon. The deviation of the resistance was negligible (R_max_/R_o _= 1.04). We also confirmed that the LED connected to the ultrathin Cu circuit (8 nm)/*a‐C*:N/PI maintained the same light intensity during folding and crumpling (Figure 5f; Movie S1, Supporting Information).

## 3 Conclusion

We have introduced a novel pathway to inhibit the corrosion of ultrathin Cu films by simply evaporating Cu onto the *a‐C*:N film substrate. Through spectroscopic and microscopic analysis, along with DFT calculations, we have uncovered the mechanism of corrosion resistance of the Cu/*a‐C*:N film. The mutual diffusion of Cu into the *a‐C*:N layer and N into the deposited Cu layer stabilized the adhesion between the two layers. This phenomenon enhanced the wettability of the deposited Cu, enabling it to form a continuous film even at a low thickness. The nanopores of the amorphous carbon and the Cu‐philic N‐containing groups in carbonaceous materials contributed to this phenomenon, distinguishing the *a*‐C:N substrate from other N‐containing substrates. Additionally, the diffusion of Cu into the *a‐C*:N substrate was propelled by the formation of thermodynamically stable C‐Cu‐N bonds, while the diffusion of N onto the Cu layer was aided by the energetically favored positioning of N on the Cu top surfaces. The significant amount of doped N in the Cu retards the adsorption of O^2−^ and OH^−^ anions into the Cu lattice, thereby halting the formation of Cu vacancies, which made the ultrathin Cu film corrosion‐resistant. We fabricated a highly flexible transparent Cu electrode by utilizing the transparency of the *a‐C*:N film, and proposed a highly stable crumpleable circuit board by simply depositing Cu on the *a‐C*:N film substrate. This approach can be a novel way to produce highly conductive flexible electrodes and circuits by utilizing contemporary industrial facilities.

## 4 Experimental Section

### Preparation of the a‐C:N, a‐C, and Graphene Films

Branched‐poly(ethyleneimine) (b‐PEI, M_W_ ≈25000, Sigma‐Aldrich) aqueous solution (40 mg mL^−1^) and D‐(+) glucose (≥99.5%, Sigma‐Aldrich) aqueous solution (0.1 M) were separately prepared. Vortex mixing and ultrasonication for 2 h to completely dissolve b‐PEI was applied. The prepared two solutions were mixed in the same volume ratio, and then sequentially filtered with a 0.45 µm‐pore poly(vinylidene fluoride) (PVDF) membrane and then a 0.2 µm‐pore PVDF membrane. A p‐doped conductive Si wafer (10 S cm^−1^) with a 300 nm SiO_2_ layer was cut into 2.5 × 2.5 cm pieces. The Si pieces were O_2_ plasma treated for 5 min at 200 W. The filtered precursor solution was spin‐coated on the Si wafer at 3000 rpm for 30 s. The coated precursor film was pre‐baked on a hot plate at 120 °C for 1 min and then put in a microwave oven (2.45 GHz microwave). The microwave irradiation conditions were 330 °C for 1 min for the 2.5 × 2.5 cm pieces and 330 °C for 3 min for the 4‐inch‐sized wafer. After microwave irradiation, the specimens were cooled in air to room temperature and washed subsequently with acetone, ethanol, and distilled water. To use quartz as a substrate for the *a‐C*:N film formation, the precursor‐coated quartz was placed on a SiO_2_/Si wafer and then subjected to the same MW irradiation conditions. The *a‐C* film with 10 nm thickness, to compare the N effect, was deposited onto the SiO_2_/Si wafer using a carbon evaporation coater (EM ACE200, Leica). The graphene‐coated SiO_2_/Si wafer (4.5 ± 0.4 kohm sq^−1^) was kindly provided by Samsung Advanced Institute of Technology, Republic of Korea.

### Cu Deposition

Cu films were thermally evaporated on the bare SiO_2_/Si wafer, *a‐C*:N/SiO_2_/Si wafer, *a‐C*/SiO_2_/Si wafer, graphene/SiO_2_/Si wafer, bare quartz, *a‐C*:N/quartz, and *a‐C*:N/PI film at a deposition rate of 2.5–3 Å s^−1^ under high vacuum (<10^−6 ^Torr). Cu patterns were deposited on each substrate through a shadow mask.

### Corrosion Test

The Cu/SiO_2_, Cu/*a‐C*:N, Cu/*a‐C*, and Cu/graphene bilayer specimens were put in a controlled humidity chamber (TH3‐PE, Jeio tech). The chamber was set to maintain 25 °C and 80% RH throughout the experiment for 14 days.

### Sample Preparation and Measurements for Electrochemical Stability Test

A heavily p‐doped conductive Si wafer (0.0015–0.005 ohm cm) was used as a substrate after etching the native oxide layer of the wafer in an aqueous HF solution (5 wt.%). For electrochemical measurement of the bare Cu (8 nm), Cu was deposited on the conductive Si wafer, and the specimen was placed on a current collector Cu plate. For the electrochemical measurement of the Cu (8 nm)/*a*‐C:N, an Au layer (50 nm) on the conductive Si wafer was deposited. After generating the *a*‐C:N layer on the Au layer, Cu was deposited on the *a*‐C:N. The specimen was placed on the current collector Cu plate. The experiments for the polarization curve were conducted using a vertical three‐electrode cell with a potentiostat instrument (PGSTAT204, Metrohm Autolab), employing a homemade electrochemical corrosion cell. An SCE electrode was employed as the reference electrode, while a graphite rod served as the counter electrode. The working electrode had an exposed area of 0.785 cm^2^. Each sample was cut to have an area of 2.89 cm^2^. The electrolyte used for the electrochemical experiments was a 3.5 wt% NaCl solution, and the experiments were carried out at room temperature without any gas flow.

The OCP was obtained by stabilizing the NaCl solution for 60 s. After the OCP measurement, the polarization curve was measured by scanning from the open circuit potential (OCP) to 100 mV in the cathodic direction and 100 mV in the anodic direction at a rate of 1 mV s^−1^. Each scan was conducted by using separate samples, and reproducibility was ensured through a minimum of three experiments. Subsequent impedance measurements were carried out from 100 kHz to 1 Hz with an amplitude of 10 mV. For the inductively coupled plasma mass spectrometry (ICP‐MS, Perkin Elmer, NexION 300S) measurements, the samples were immersed in 3.5 wt.% NaCl solution (50 mL) at room temperature for 10 min. After the immersion process, the samples were taken out, and ICP‐MS measurements were performed for each solution.

### Characterizations

The optical microscope (OM) images were obtained using an optical microscope (BX51M, Olympus), and the sheet resistance was measured at 5 points in all samples using a four‐point probe (CMT‐100S, Advanced Instrument Technology). The optical transmittance spectra were obtained using a UV–Vis spectrophotometer (V‐770, JASCO). X‐ray photoelectron spectroscopy (XPS) and depth XPS (Nexsa G2, Thermo Fisher) measurements were carried out with monochromatic Al of 12 kV and 10 mA X‐ray sources. The surface topography and thickness were measured by atomic force microscopy (AFM) (Dimension Icon, Bruker). High‐resolution transmission electron microscope (HRTEM) images were acquired with JEM‐2200FS (JEOL) at an acceleration voltage of 200 kV, together with its EELS mapping and SAED. A focused ion beam (FIB) (Helios NanoLab 450, FEI and Helios 600, FEI) was used to prepare a specimen for cross‐sectional HRTEM. The Raman spectra were obtained by a micro‐Raman spectrometer (Alpha 300 RA, Witec), with the wavelength and the spot size of the laser excitation were 532 nm and 1.0 µm, respectively. The X‐ray diffraction (XRD) patterns were collected with D/MAX‐2500/PC (Rigaku) and the lattice constant measurements were referred from JCPDS card no. 851326 for Cu, no. 782076 for Cu_2_O, no. 800656 for Cu(OH)_2_, and no. 801917 for CuO.

### Fabrication of the Transparent Crumpleable Cu/a‐C:N/PI Electrode

To prepare the flexible substrate, polyimide (PI) solution (Polyimide P84, Evonik) was spin‐coated (3000 rpm for 60 s) on the *a‐C*:N/SiO_2_. The PI‐coated *a‐C*:N/SiO_2_ was pre‐baked on a hot plate at 80 °C for 10 min to evaporate the remaining solvent, and then cured at 280 °C for 2 h. The cured PI film (15 µm)/*a‐C*:N/SiO_2_ was immersed in a water bath. The PI/*a‐C*:N was completely peeled off from the SiO_2_/Si wafer when cut along the edges with a razor blade under water.

### Deformability Test

For the bending test of the Cu mesh (20 nm)/*a‐C*:N/PI, the test was conducted with UMP (Teraleader Co. Ltd., Korea) at a 2 mm bending radius at 1 V bias. For crumpling and folding test of the Cu circuit (8 nm)/*a‐C*:N/PI, the LEDs attached to the Cu circuit/*a‐C*:N/PI film were connected to a function generator (33220A Function/arbitrary waveform generator, 20 MHz, Keysight) at 2 V (DC bias), and then crumpling and folding were applied while measuring the luminescence intensity.

### Classical Molecular Dynamics (CMD) Calculations

It was performed with the Large‐scale Atomic/Molecular Massively Parallel Simulator (LAMMPS) code by using the reactive force field (ReaxFF) interatomic potential.^[^ ^81^, ^82^ ^]^ NVT ensemble calculations were carried out to model the nitrogen‐doped amorphous carbon (*a‐C*:N) system. The Nose/Hoover thermostat was used to control the temperature and the damping constant was set to 0.1 ps. The density of *a‐C*:N and time step were set to 2.0 g cm^−3^ and 0.1 fs, respectively. The distribution of atoms in *a‐C*:N were randomized at 2000 K for 10 ps and melted at 1000 K for 10 ps. After melting, the system was quenched from 1000 K to 300 K, with a cooling rate of 70 K ps^−1^. The quenched structures at 300 K for 10 ps were further equilibrated.

### Density‐Functional Theory (DFT) Calculations

It was calculated using the Vienna Ab initio Simulation Package (VASP) code employing the projector augmented wave (PAW) method.^[^ ^83^, ^84^, ^85^ ^]^ The kinetic cutoff energy for the plane wave expansion was set to 500 eV. The semilocal PBE functional due to Perdew, Burke, and Ernzerhof (with D3 dispersion corrections to consider van der Waals interactions) was used.^[^ ^86^ ^]^ For the bulk calculations, a 4 × 4 × 4 supercell, and a Γ centered k‐point grid spacing of 0.15  Å^−1^ were used. For the large *p*(5 × 5) surface models, the Brillouin zone only at the Γ point was sampled. The vacuum region was set to at least 15 Å and a dipole correction was applied to avoid unphysical interactions between neighboring slabs in the z‐directions.

The adsorption energy of the atom on the slab model, *E*_ad_ was calculated as

(1) $$E_{\mathrm{ad}}=E_{\mathrm{slab}/\mathrm{ads}}\;\;-\;E_{\mathrm{slab}}-E_{\mathrm{ads}}$$

where *E*_slab/ads_, *E*_slab_, and *E*_ads_ were the total energies of the total system, the surface slab model, and the adsorbate (which is either Cu or O) in question, respectively. For *E*_ads_, the total energy (per atom) of bulk Cu and that of molecular O_2_, respectively were considered.

The formation energy of Cu vacancy, *E_vac_* was calculated as

(2) $$E_{vac}=E_{N-1}\;\;-\;\frac{N-1}{N}E_{N}$$

where *N*, *E_N_*, and *E*_*N* − 1_ were the number of the Cu atoms in the bulk supercell, the total energy of the bulk Cu supercell without the Cu vacancy, and the total energy of that with one Cu vacancy, respectively.

## Conflict of Interest

The authors declare no conflict of interest.

## Supporting information

Supporting Information

Supporting Movie S1

## Data Availability

The data that support the findings of this study are available from the corresponding author upon reasonable request.
