# Supplementary material for: Corrosion‐Resistant Ultrathin Cu Film Deposited on N‐Doped Amorphous Carbon Film Substrate and Its Use for Crumpleable Circuit Board
Source: Adv Sci (Weinh). 2024 Aug 29;11(40):2403587. doi: 10.1002/advs.202403587 (PMC11516104; doi:10.1002/advs.202403587)
Supplement: Supplementary file 1 — Supporting Information [file ADVS-11-2403587-s001.pdf]

ADVANCED SCIENCE
Open Access

Supporting Information

for *Adv. Sci.*, DOI 10.1002/advs.202403587

Corrosion-Resistant Ultrathin Cu Film Deposited on N-Doped Amorphous Carbon Film Substrate and Its Use for Crumpleable Circuit Board

Chae-Eun Shim, Sangseob Lee, Minsik Kong, Ik-Soo Kim, Jaeik Kwak, Woosun Jang, Se-Young Jeong, Dong Wook Kim, Aloysius Soon* and Unyong Jeong**

Corrosion-Resistant Ultrathin Cu Film Deposited on N-Doped Amorphous Carbon Film Substrate and Its Use for Crumpleable Circuit Board

Chae-Eun Shim, Sangseob Lee, Minsik Kong, Ik-Soo Kim, Jaeik Kwak, Woosun Jang, Se-Young Jeong, Dong Wook Kim, Aloysius Soon*, and Unyong Jeong**

C.-E. Shim, M. Kong, I.-S. Kim, J. Kwak, and Prof. U. Jeong
Department of Materials Science and Engineering, Pohang University of Science and Technology (POSTECH), Pohang, 37673, Republic of Korea
*E-mail: ujeong@postech.ac.kr

S. Lee and Prof. A. Soon
Department of Materials Science and Engineering and Center for Artificial Synesthesia Materials, Yonsei University, Seoul, 03722, Republic of Korea.
*E-mail: aloysius.soon@yonsei.ac.kr

Prof. W. Jang
Department of Integrated Science and Engineering Division, Underwood International College, Yonsei University, Incheon, 21983, Republic of Korea

D. W. Kim
Physical Intelligence Department, Max Planck Institute for Intelligent Systems, Stuttgart, 70569, Germany.
*E-mail: dongwkim@is.mpg.de

Prof. S.-Y. Jeong
Gordon Center for Medical Imaging, Department of Radiology, Massachusetts General Hospital and Harvard Medical School, Boston, MA, 02114, USA.

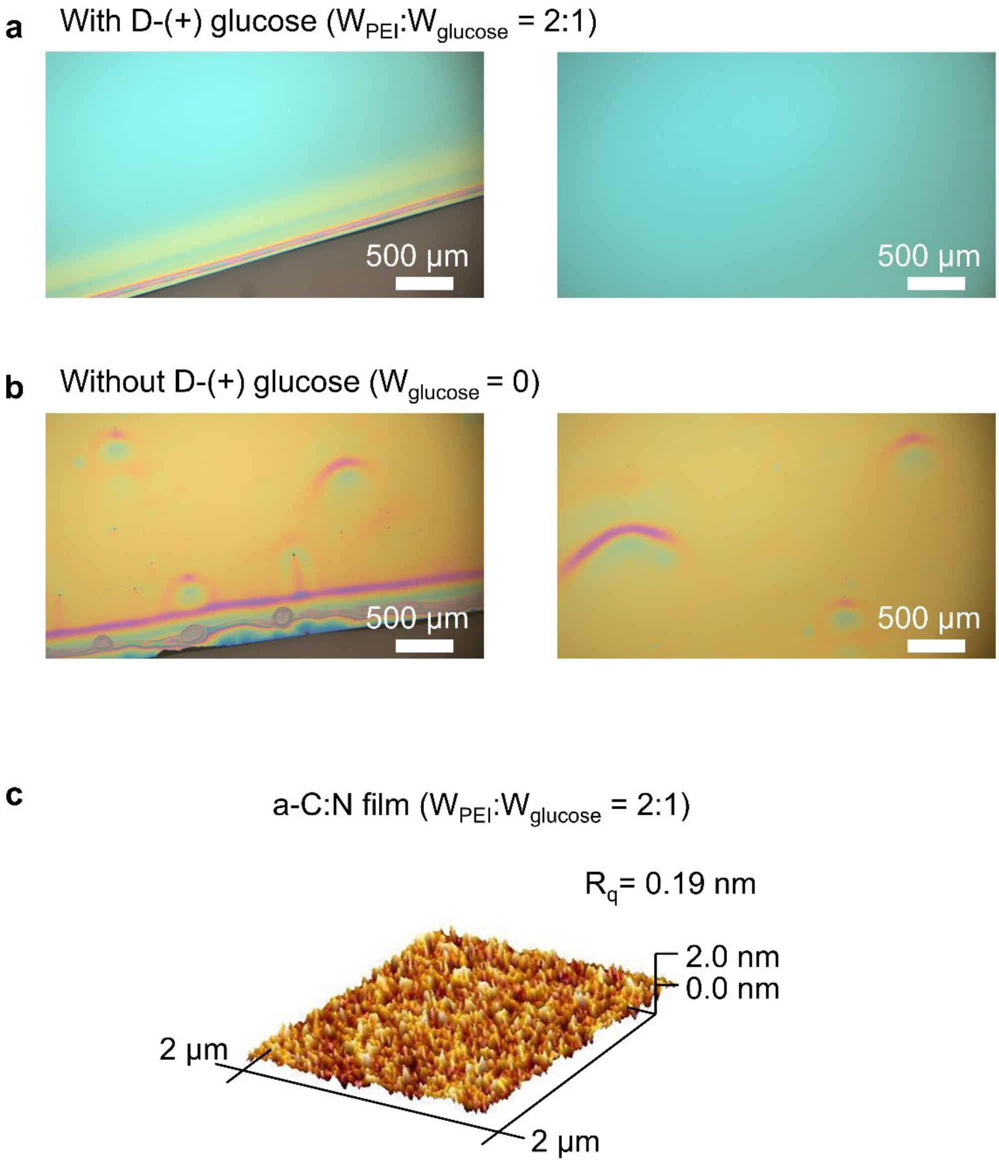

Figure S1. a, b) The optical microscopy (OM) images of the baked precursor polymer film; (a) with D-(+) glucose (W_{PEI}:$W_{glucose}$ = 2:1), (b) without D-(+) glucose ($W_{glucose}$ = 0). c) Atomic force microscopic (AFM) image and surface roughness (R_q) of the *a-C*:N film (PEI:D-(+) glucose= 2:1). The R_q is measured in a 2 µm × 2 µm area.

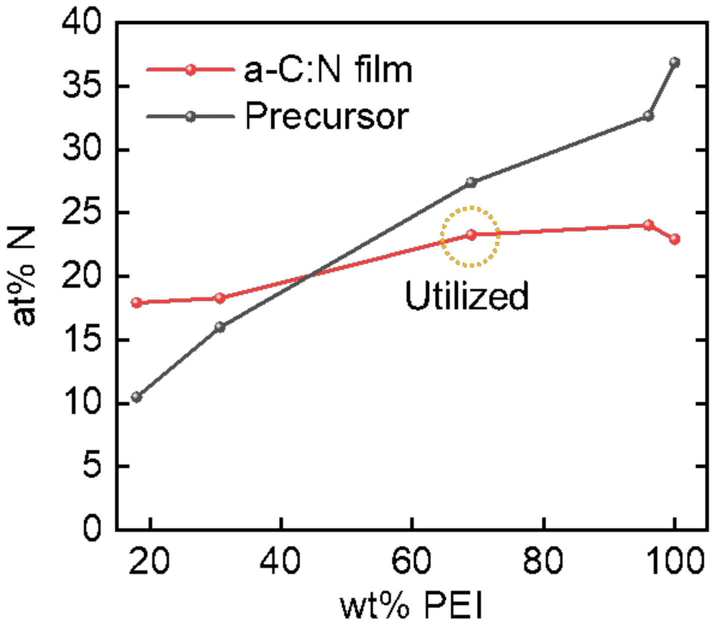

Figure S2. Theoretical atomic percentage of N (at% N) in the b-PEI/ D-(+) glucose polymer film (precursor) and experimentally measured X-ray photoelectron spectroscopy (XPS) of at% N in the *a-C*:N film varied by the weight percentage of b-PEI (wt% PEI). The *a-C*:N film with 69 wt% PEI (W_{PEI}:$W_{glucose}$ = 2:1) was utilized in this work (The dotted yellow circle one).

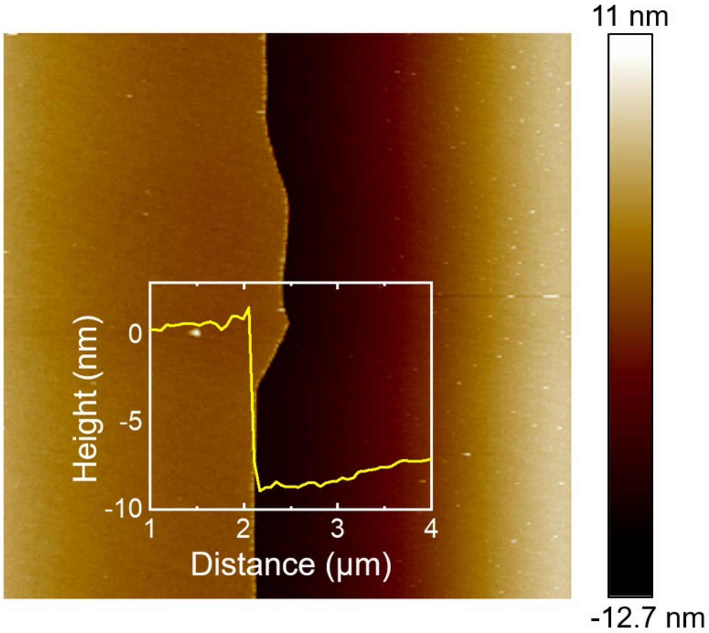

Figure S3. AFM image showing the height profile of *a-C*:N film and its surface uniformity.

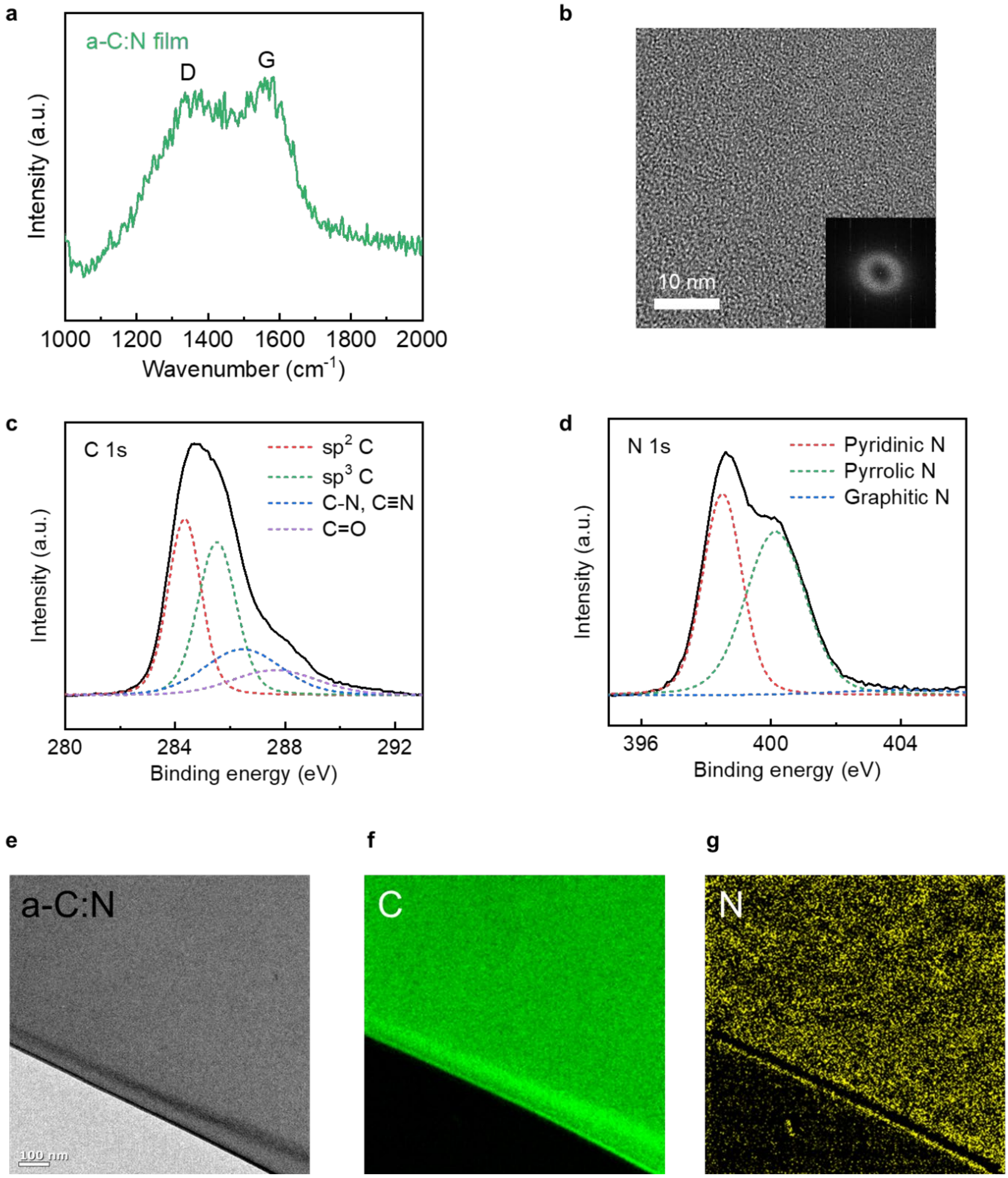

Figure S4. a) Raman spectrum of *a-C*:N film showing D and G peaks. b) High-resolution transmission electron microscopy (HRTEM) image of *a-C*:N film and the corresponding fast Fourier transform image (inset) showing its amorphousness. c-d) C 1s and N 1s spectra from XPS of *a-C*:N film. e-g) TEM image and its electron mapping (C and N) from electron energy loss spectroscopy (EELS) images of *a-C*:N film.

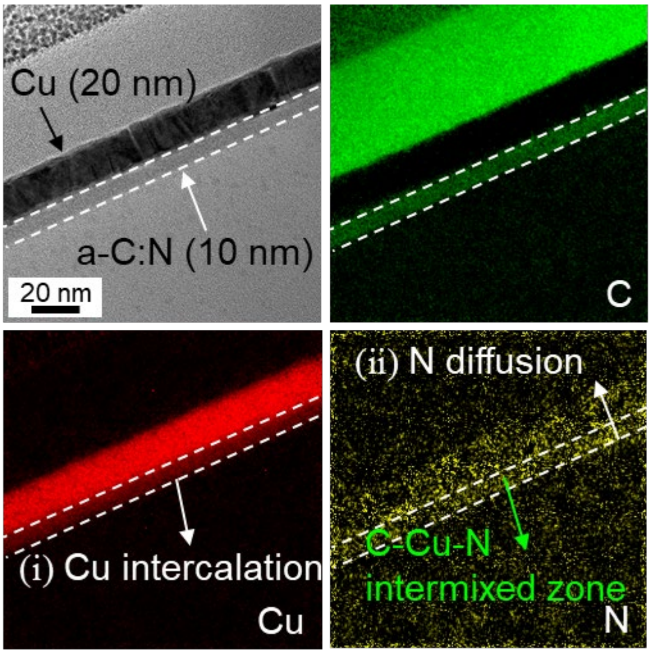

Figure S5. Cross-sectional TEM image and its EELS mapping for the Cu (20 nm)/*a*-*C*:N bilayer indicating Cu intercalation within the *a*-*C*:N film (i) and N diffusion to the Cu surface with the C-Cu-N intermixed zone (ii).

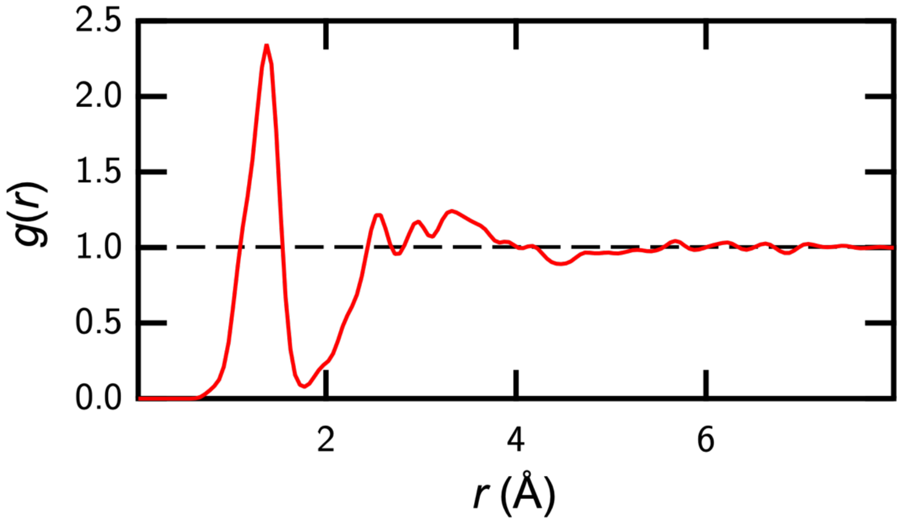

Figure S6. Radial distribution function (RDF, g(r)) for the bulk *a*-*C*:N.

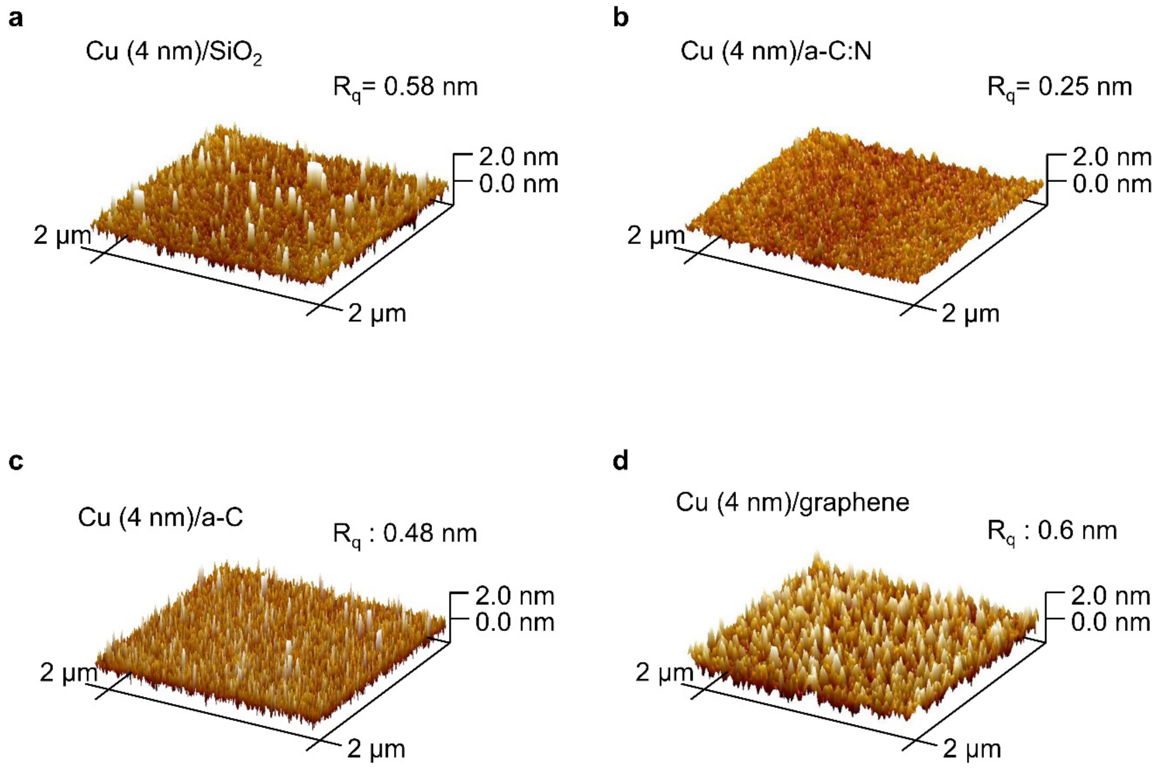

Figure S7. a-d) AFM images and their surface roughness (R_q) of Cu (4 nm)/SiO_2 (a), Cu (4 nm)/*a-C*:N (b), Cu (4 nm)/*a-C* (c), and Cu (4 nm)/graphene (d). The R_q is measured in a 2 µm × 2 µm area.

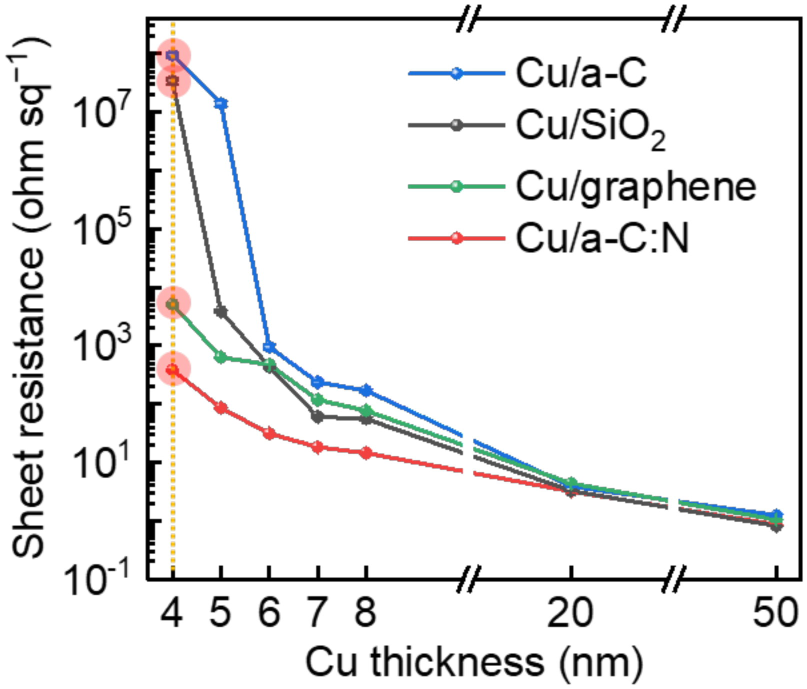

Figure S8. Changes in the sheet resistance of Cu/SiO_2, Cu/*a-C*, Cu/graphene, and Cu/*a-C*:N as a function of the Cu thickness (t_{Cu}). The yellow dashed line is indicated at t_{Cu} = 4 nm.

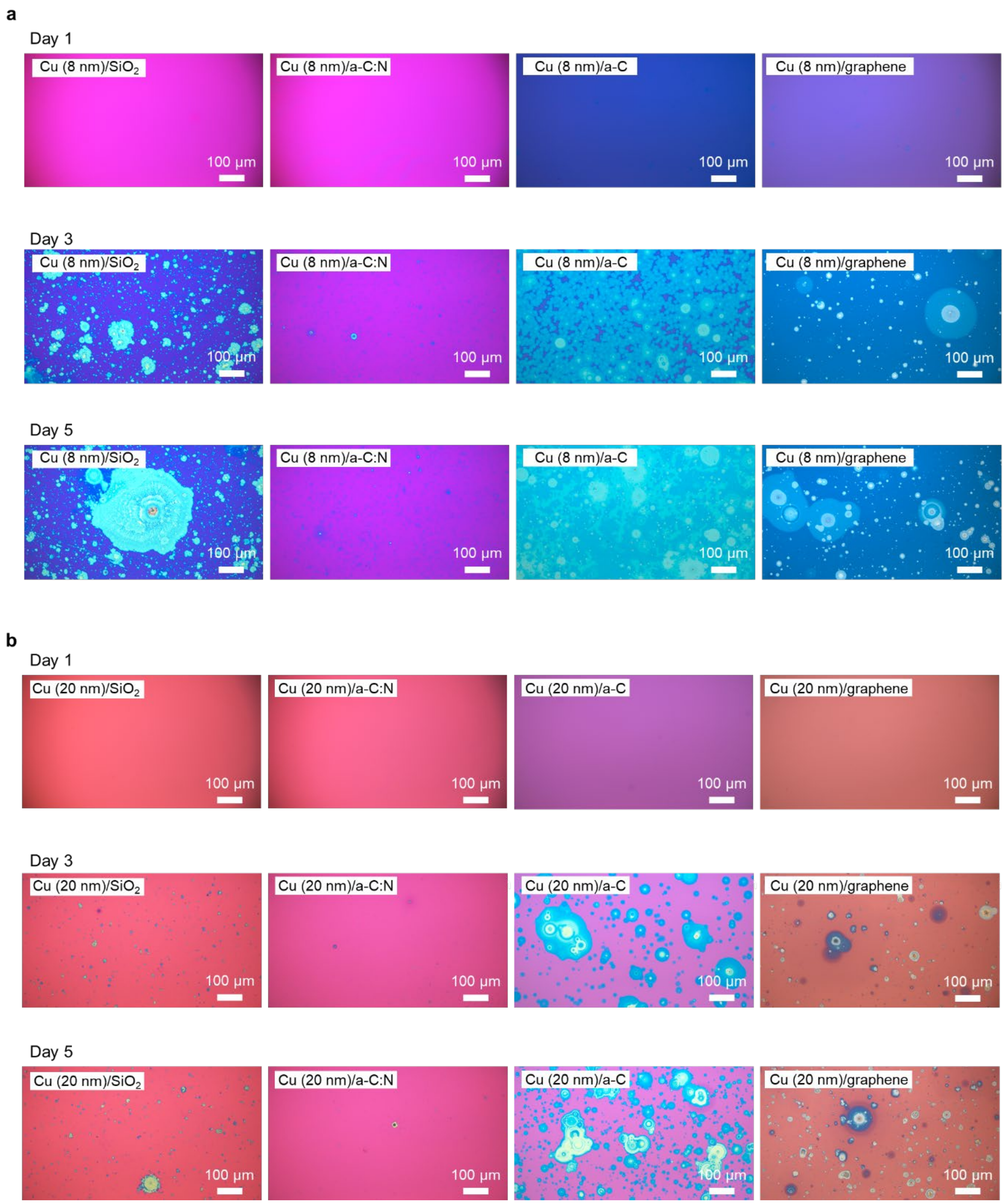

Figure S9. Changes in OM images of corroded Cu film on the SiO_2, *a-C*:N, *a-C*, and graphene substrates observed on day 1, day 3, and day 5 under humid conditions (25 °C, 80% RH); (a) 8 nm-thick Cu layer, (b) 20 nm-thick Cu layer.

Table S1. Changes in the sheet resistance (R_s) of the Cu films (8 nm and 20 nm) on SiO_2 and *a-C*:N substrates during the corrosion test (25 °C, 80% RH) for 15 days, as shown in Figure 2d.

Sample	Day 0	Day 1	Day 2	Day 3	Day 4	Day 5	Day 6	Day 7
Cu (8 nm) /SiO_2	61.3 ± 29.7	113.2 ± 68.8	92.2 ± 24.1	140.2 ± 74.6	132.4 ± 65.1	142.7 ± 52	130.1 ± 25.6	272.4 ± 180.5
Cu (8 nm) /*a-C*	40.2 ± 7.9	76.1 ± 28.8	1474.4 ± 1325.1	32.3 ± 11.4M	104 ± 8.1 M	71.1 ± 33.2 M	105 ± 19.6 M	137 ± 24.7 M
Cu (8 nm) /*a-C*:N	12.4 ± 0.3	15 ± 0.12	15 ± 0.14	15.9 ± 0.4	16.7 ± 0.4	16.5 ± 0.4	17.2 ± 0.6	19.2 ± 0.8
Cu (20 nm) /SiO_2	3.7	3.8	3.8	3.9	3.7	3.8	3.8	3.8
Cu (20 nm) /*a-C*	3.5	4.1 ± 0.1	4.3 ± 0.1	27.5 ± 3.83	21.2 ± 3.9	44.1 ± 16.8	62 ± 39	61.9 ± 37.2
Cu (20 nm) /*a-C*:N	3.7	3.5	3.7	3.6	3.7	3.7	3.9	3.8

Sample	Day 8	Day 9	Day 10	Day 11	Day 12	Day 13	Day 14
Cu (8 nm) /SiO_2	258.6 ± 160.4	297.8 ± 53.1	305.2 ± 104	309 ± 62.4	312.7 ± 52.4	7.7 ± 4.8 M	23.3 ± 14.5 M
Cu (8 nm) /*a-C*	180 ± 25.3 M	251 ± 56 M	291 ± 63 M	205 ± 59 M	232 ± 28.7 M	297 ± 59.2 M	201 ± 19.1 M
Cu (8 nm) /*a-C*:N	23 ± 0.7	22 ± 0.4	23.4 ± 0.2	24 ± 0.6	23 ± 0.3	24 ± 0.4	26 ± 0.6
Cu (20 nm) /SiO_2	3.9	3.8	3.9	3.9	4	4.2 ± 0.1	4.3 ± 0.1
Cu (20 nm) /*a-C*	332 ± 323	392.1 ± 384	11 ± 10.9 k	3.1 ± 3.1 M	6.1 ± 6.1 M	22.8 ± 22.8 M	15.2 ± 15.2 M
Cu (20 nm) /*a-C*:N	3.7	3.8	3.7	3.6	3.7 ± 0.1	3.8	3.9

(Unit: ohm sq^{-1})

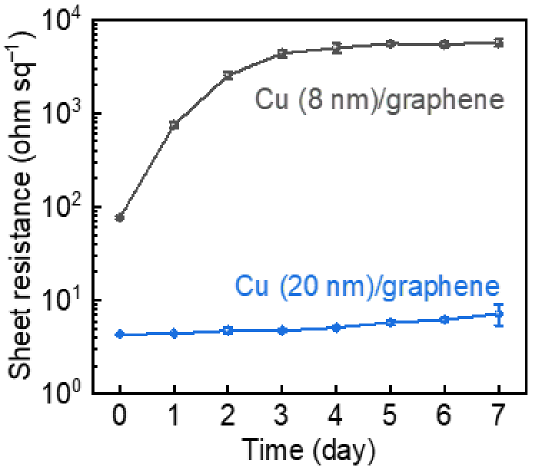

Figure S10. The changes in sheet resistance (R_s) of Cu film (8 nm and 20 nm) deposited on graphene for 7 days under humid conditions (25 °C, 80% RH).

Table S2. Changes in the sheet resistance (R_s) of the Cu films (8 nm and 20 nm) on the graphene substrates during the corrosion test (25 °C, 80% RH), as shown in Figure S10.

Sample	Day 0	Day 1	Day 2	Day 3	Day 4	Day 5	Day 6	Day 7
Cu (8 nm)/ Graphene	76.6 ± 2	747.3 ± 53.4	2527.6 ± 217.2	4355.6 ± 365.9	4985.4 ± 591.3	5540 ± 213.6	5449.2 ± 320.9	5661.4 ± 512.8
Cu (20 nm)/ Graphene	4.3	4.4	4.8 ± 0.3	4.7 ± 0.1	5.1 ± 0.1	5.8 ± 0.3	6.2 ± 0.3	7.2 ± 1.8

(Unit: ohm sq^{-1})

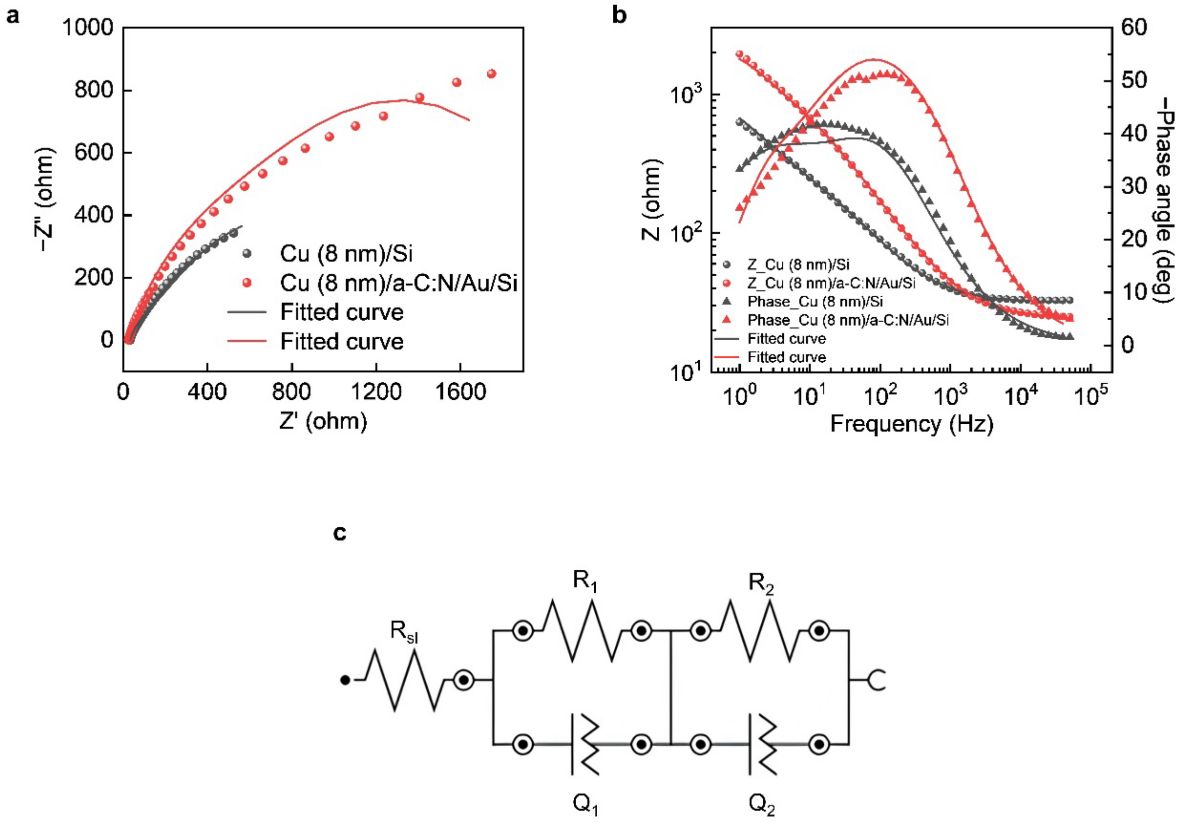

Figure S11. Nyquist (a) and Bode (b) plots of Cu (8 nm)/Si and Cu (8 nm)/*a-C*:N/Au/Si in a 3.5 wt% NaCl solution under OCP conditions. The equivalent electrical circuit (EEC) model (c) used for fitting the EIS experimental data. Q_1 and Q_2 indicate the constant phase elements (CPEs).

Table S3. Equivalent circuit parameters for EIS measurement of Cu (8 nm)/Si and Cu (8 nm)/*a-C*:N/Au/Si in a 3.5 wt% NaCl solution under OCP conditions.

**Parameter**	**Cu (8 nm)/Si**	**Cu (8 nm)/*a-C*:N/Au/Si**
R_{sl} (ohm)	31.4	24.18
R_1 (ohm)	1373.7	1012.4
CPE (Q_1) (μMho s^N)	3.77×10^{-4}	5.88×10^{-5}
N	0.67	0.73
R_2 (ohm)	99.41	1120.2
CPE (Q_2) (μMho s^N)	2.18×10^{-4}	1.05×10^{-4}
N	0.74	1
χ^2	6.03×10^{-2}	6.99×10^{-2}

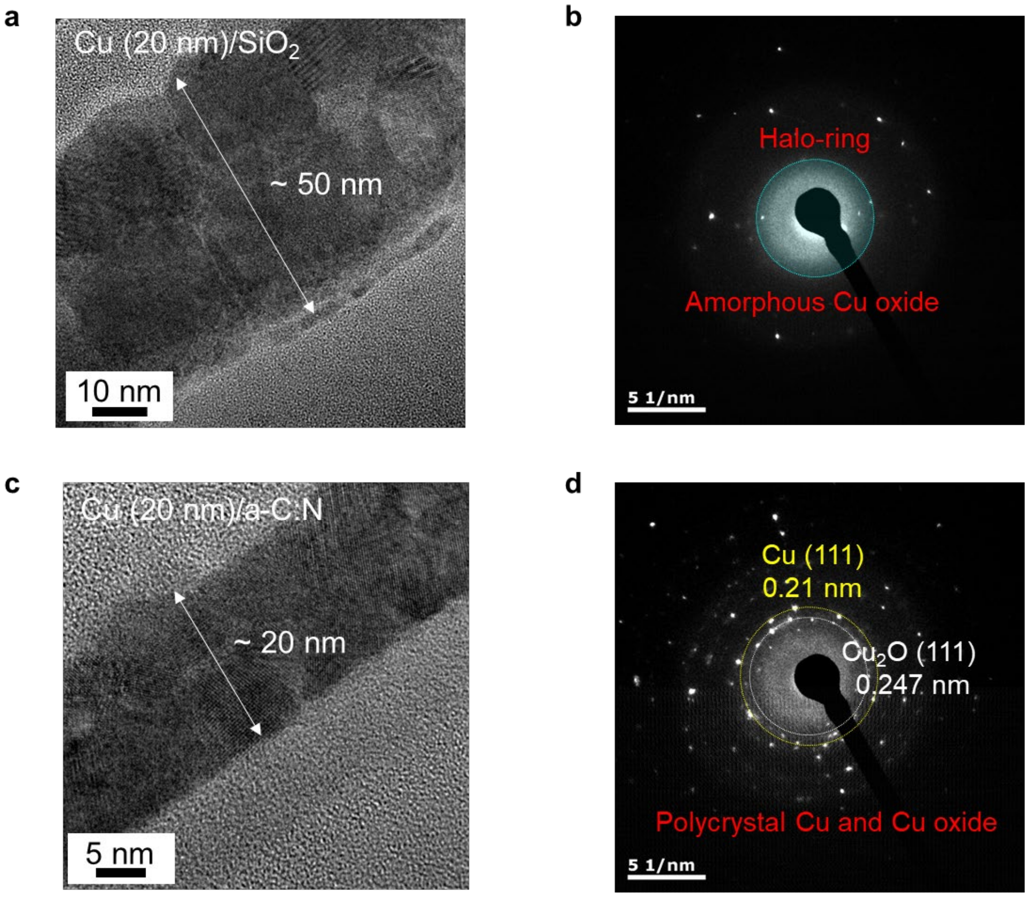

Figure S12. a, b) Cross-sectional TEM images (magnification of 400k) (a) and selected area electron diffraction (SAED) patterns (b) of the oxidized Cu (20 nm)/SiO_2. c, d) Cross-sectional TEM images (magnification of 600k) (c) and selected area electron diffraction (SAED) patterns (d) of the oxidized Cu (20 nm)/*a*-*C*:N/SiO_2.

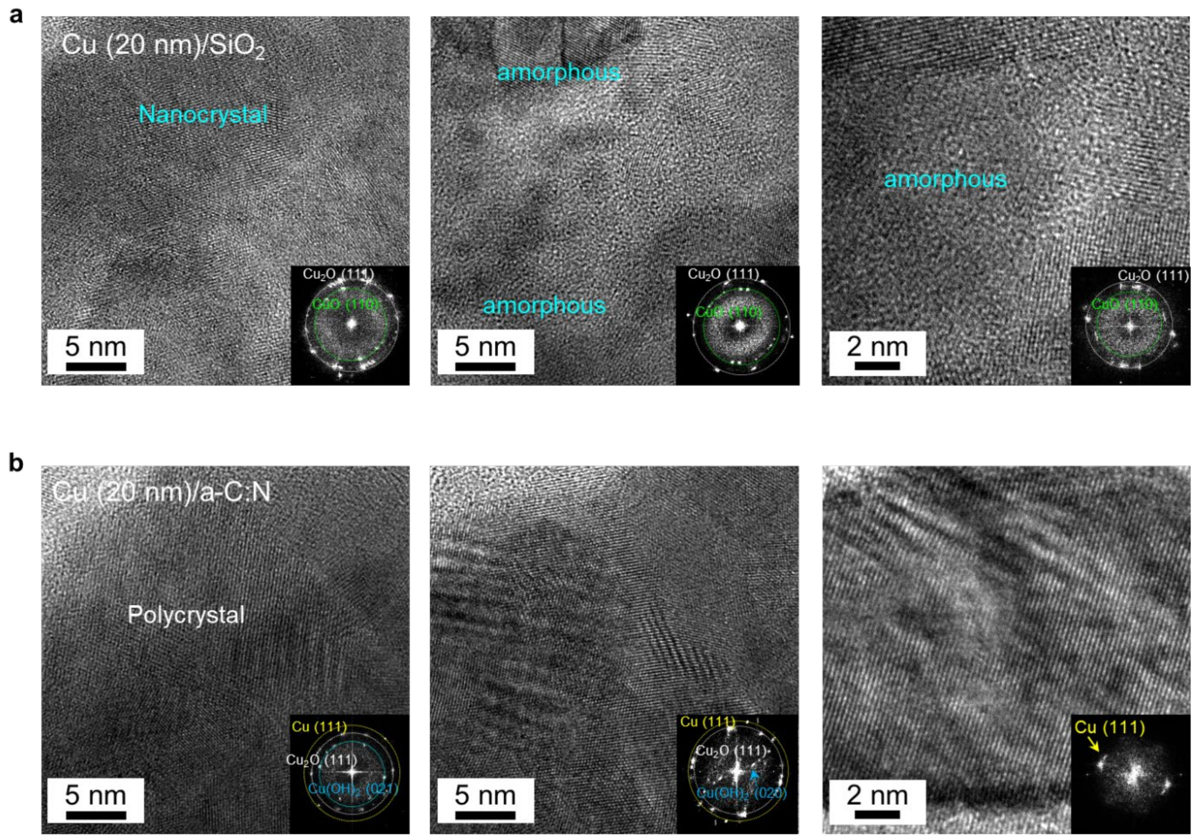

Figure S13. Enlarged cross-sectional TEM images with their FFT image for the oxidized Cu (20 nm) deposited on SiO_2 (a) and the *a-C*:N film (b).

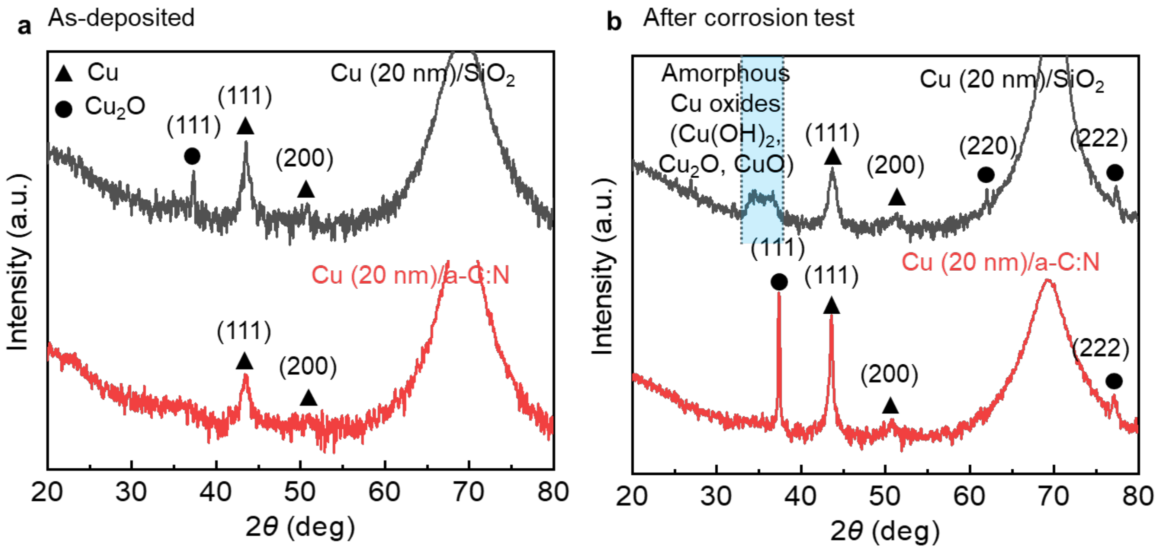

Figure S14. a, b) X-ray diffraction (XRD) patterns of the Cu film (20 nm) on the SiO_2 substrate and *a-C*:N film before (as-deposited) (a) and after corrosion test (b) for 7 days under humid conditions (25 °C, 80% RH)

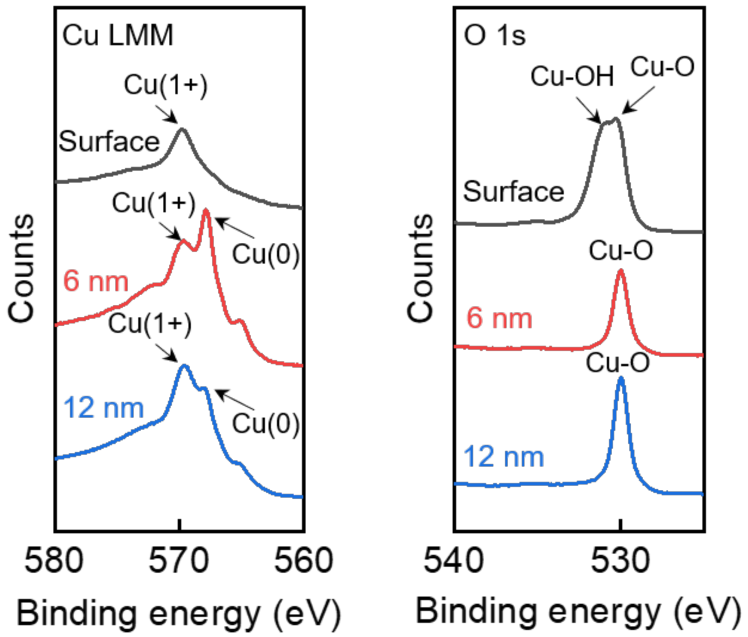

Figure S15. Depth XPS profile analysis in the Cu LMM and O 1s of corroded Cu film (20 nm) on *a-C* substrate.
